# Bimodal iontronic skins powered by edge intelligence for real-time collaborative interaction

**DOI:** 10.1093/nsr/nwag111

**Published:** 2026-02-14

**Authors:** Zhibin Li, Junli Shi, Xinxing Chen, Xingxing Chen, Ping Yang, Hanxiang He, Yuquan Leng, Xintao Huan, Han Hu, Chenglong Fu, Taihong Wang, Chuan Fei Guo

**Affiliations:** Department of Electronic and Electrical Engineering, Southern University of Science and Technology, Shenzhen 518055, China; Department of Materials Science and Engineering, Southern University of Science and Technology, Shenzhen 518055, China; The Key Laboratory of Image Processing and Intelligent Control and the Hubei Key Laboratory of Brain-inspired Intelligent Systems, School of Artificial Intelligence and Automation, Huazhong University of Science and Technology, Wuhan 430074, China; Department of Mechanical and Energy Engineering, Southern University of Science and Technology, Shenzhen 518055, China; Department of Materials Science and Engineering, Southern University of Science and Technology, Shenzhen 518055, China; Department of Mechanical and Energy Engineering, Southern University of Science and Technology, Shenzhen 518055, China; School of Information and Electronics, Beijing Institute of Technology, Beijing 100081, China; Department of Mechanical and Energy Engineering, Southern University of Science and Technology, Shenzhen 518055, China; School of Cyberspace Science and Technology, Beijing Institute of Technology, Beijing 100081, China; School of Information and Electronics, Beijing Institute of Technology, Beijing 100081, China; Department of Mechanical and Energy Engineering, Southern University of Science and Technology, Shenzhen 518055, China; Department of Electronic and Electrical Engineering, Southern University of Science and Technology, Shenzhen 518055, China; Department of Materials Science and Engineering, Southern University of Science and Technology, Shenzhen 518055, China

**Keywords:** electronic skin, iontronic sensor, embodied intelligence, human–robot interaction, deep learning

## Abstract

Real-time sensing and processing of large-scale tactile information are crucial for enhancing the compliant interaction of embodied robots, especially in collaborative systems. However, existing robotic skin systems are limited by latency in high-throughput signal readout and intelligent reasoning, making robust real-time interaction challenging. Here, we present a flexible bimodal skin powered by edge intelligence, enabling real-time sensory perception, decision-making and actuation based on large-area coverage. The modular bimodal skin integrates pressure and temperature sensors, providing full coverage on robotic arm with over 768 pressure and 75 temperature sensor units. A rapid, crosstalk-free readout interface is implemented using a frequency-encoding architecture. Furthermore, we develop a lightweight deep learning framework that enables real-time autonomous decision-making for the bimodal skin at the edge device. We demonstrate that our system facilitates smooth, adaptive interaction for individuals with mobility impairments, even under complex or emergency conditions. This technology opens a promising avenue for real-time perception and interaction in human-centered embodied robotics.

## Teaser

This work presents a large-area bimodal iontronic skin integrated with lightweight edge-intelligence, enabling real-time tactile perception, decision-making, and adaptive human–robot collaboration for safe and compliant embodied robotic interaction.

## INTRODUCTION

The rapid advancement of artificial intelligence and sensing technologies has driven significant progress in embodied robotics [1–3]. Compliant interactions driven by the accurate understanding of human intent is one of the ultimate goals of human-centered robotics, particularly in collaborative systems [4,5]. Human–robot collaboration requires real-time unification of sensory perception, decision-making and actuation within cohesive robotic architectures, to provide responsive assistance and mutual adaptation [3,6–10]. However, accurately perceiving and interpreting human intent in real time—particularly under complex and variable interaction patterns—remains a significant challenge [11–17].

In collaborative human–robot scenarios, sudden environmental changes often lead to instinctive and unstructured physical interactions [4,18–20]. Accordingly, artificial skins for robotic surface coverage have been widely reported, endowing robots with multimodal sensory capabilities to respond to diverse interaction modes (such as grasping, sliding and twisting) or multiple physical parameters (distributed pressure and temperature) [21–27]. Tactile-based robotic perception and feedback provide an effective pathway toward safer and more compliant human–robot collaboration [28,29]. However, as the number of sensing modalities and sensor units increases, large-scale sensor integration systems face critical bottlenecks of sensing crosstalk [30,31] and readout latency [23].

Moreover, deep learning-enhanced intelligent sensing systems have been extensively demonstrated to improve the compliance and robustness of human–robot interactions [32,33]. Most previous sensing systems collect signals and perform deep-learning-based post-processing either offline (even days after data acquisition) or via cloud computing, which significantly increases latency [34]. Notably, intelligent models embedded at the sensor edges can interpret user commands and environmental cues in real time based on diverse sensory signals [35], providing rapid execution feedback. This local processing significantly reduces latency and enhances responsiveness, which is crucial for embodied robotic collaboration. However, the substantial increase in sensing signal volume and the growing complexity of deep learning models limit the real-time decision-making and dynamic feedback in edge systems [36,37].

**Figure 1.**
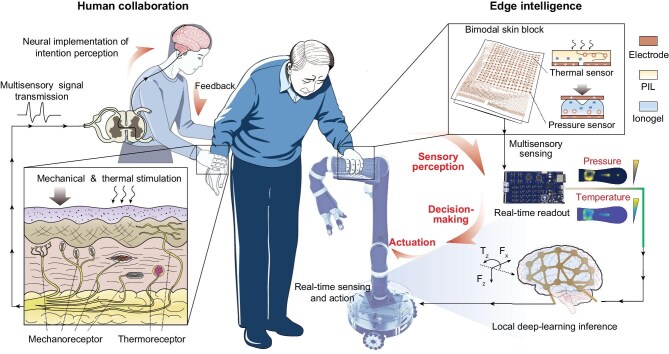
Overview of the flexible bimodal skin powered by edge intelligence for large-area robotic coverage to enhance real-time collaborative interaction. This tactile skin system is deployed on edge-embodied robots, enabling real-time sensory perception, decision-making and actuation based on large-area coverage.

Here, we introduce a flexible bimodal skin powered by edge intelligence that enables real-time sensory perception, decision-making and actuation based on large-area coverage (Fig. 1). This modular bimodal skin integrates pressure and temperature sensors, providing full coverage on a robotic arm with over 768 pressure and 75 temperature sensor units. The readout circuitry is embedded with a frequency encoding architecture, ensuring rapid, crosstalk-free parallel readout of the large-scale iontronic array. Furthermore, we develop a lightweight deep learning framework that enables the bimodal skin to interpret human interaction intent in real time on edge devices, facilitating autonomous decision-making. We further demonstrate that the system operates reliably in robot-assisted collaborative scenarios, providing effective support for individuals with mobility impairments and enhancing their real-time collaborative capabilities. This technology opens a promising path for human-centered embodied robots in real-time perception and interaction.

## RESULTS

### Fabrication, principle and properties of the pressure/temperature bimodal skin

**Figure 2.**
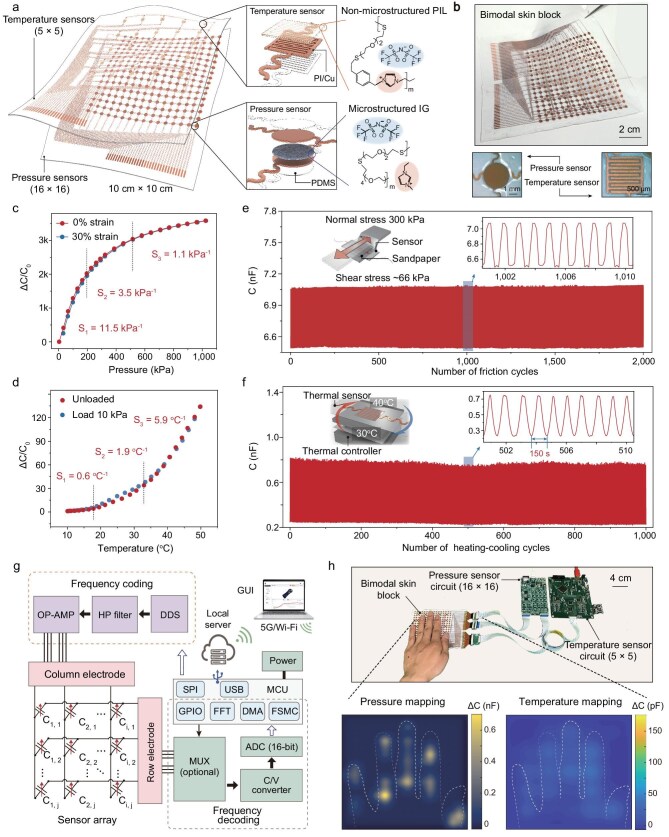
Fabrication, materials and properties of the bimodal skin. (a) Diagram illustration of the iontronic skin, showing two types of sensors: temperature sensor and pressure sensor. The active material in the temperature sensor is a flat PIL, and that in the pressure sensor is a microstructured ionogel (IG). (b) Optical image of the bimodal iontronic skin. (c) Normalized change in capacitance as a function of pressure of the pressure sensor. (d) Normalized change in capacitance as a function of temperature of the temperature sensor. (e) Signal variation of the pressure sensor under cyclic tangential loading at 66 kPa (using #500 sandpaper with a friction coefficient of 0.22). The sensor demonstrates excellent cycling stability due to its strong interfacial adhesion. (f) Signal variation of the temperature sensor under cyclic temperature fluctuations in the range of 30°C–40°C. The sensor exhibits outstanding cycling stability, attributed to the purely physical nature of the electrical double-layer capacitance and the entropy-driven process of ion dissociation and diffusion at different temperatures. (g) Back-end interface design based on frequency-encoding architecture. DDS, direct digital synthesizer; OP-AMP, operational amplifier; HP filter, high pass filter; MUX, multiplexer; C/V converter, capacitance-to-voltage converter; ADC, analog to digital converter; FSMC, flexible static memory controller; DMA, direct memory access; FFT, fast Fourier transform; MCU, microcontroller unit. (h) Pressure and temperature response of the bimodal skin when touched by a palm.

Both pressure and temperature sensors in the skin are iontronic devices; the pressure sensors use a microstructured ionogel layer and the temperature sensors use a flat poly-ionic liquid (PIL) layer as the active materials. Both the ionogel and the PIL (detailed preparation of monomers is shown in Fig. S1) were synthesized via a photo-initiated thiol-ene click reaction (principles and characterization of the material are provided in Figs S2–S4). The capacitive signals of our devices derive from the electric double layers (EDLs) formed at the interfaces between the ionic (ionogel or PIL) and electronic conductors (i.e. polyimide–copper electrode). The capacitance density of EDLs is 4–5 orders of magnitude higher than that of traditional dielectric-based capacitors because of the atomic-scale charge separation in EDLs [38], enabling excellent sensing properties in iontronic skins. As shown in Fig. 2a, each modular bimodal skin block consists of a layer of temperature sensors (5 × 5 grid) stacked on top of a layer of pressure sensors (16 × 16 grid), covering an area of 10 cm × 10 cm. In the human palm, two-point discrimination for mechanical stimulation is 6–10 mm [39], and the spacing of our pressure sensor array (∼6 mm) is sufficient to achieve effective tactile perception. For the pressure sensors, the contact area between the microstructured surface of the ionogel and the electrodes changes with applied pressure, leading to the variation in EDL capacitance for pressure sensing. For the temperature sensors, the change in temperature alters the ionic conductivity of PIL and thus the EDL capacitance for temperature sensing. The photograph of the skin block is shown in Fig. 2b. Our sensing units are interconnected via serpentine lines, which can offer high stretchability, facilitating conformal attachment to curved surfaces and minimizing mechanical interference [40–42]. In our skin, all interfaces of the sensors were chemically bonded (see Note S1 in the Supplementary data and Fig. S5) to ensure high interfacial stability [43,44]. The details of the fabrication of the iontronic skin are discussed in Note S2 and Figs S6 and S7.

Our iontronic skins exhibit ultrahigh sensitivity to both pressure and temperature. Sensitivity is defined as the normalized change in capacitance over the change in pressure/temperature, expressed as δ(*C*/*C*_0_)/δ*P* for the pressure sensor and δ(*C*/*C*_1_)/δ*T* for the temperature sensor, where *C*_0_ is the initial capacitance value of the pressure sensor before loading, *C*_1_ is the capacitance of the temperature sensor at 10°C, and *P* and *T* represent instantaneous pressure and temperature, respectively. The microstructure (Fig. S8) used in the pressure sensor can provide both high sensitivity and working range [45]—even at an ultrahigh pressure of 1000 kPa, the sensitivity is still as high as 1.1 kPa^–1^ (Fig. 2c, Fig. S9a). Meanwhile, the serpentine interconnections and relatively high stiffness of the sensing units provide strain-insensitivity under a tensile strain up to 30% (Table S1).

The temperature sensor contains a flat PIL layer sandwiched between two electrodes ensuring that it does not respond to pressure. As temperature increases, the heat excites the dissociation of ion pairs and the migration of the dissociated ions to the iontronic interface, generating an increased EDL capacitance. The PIL-based temperature sensor exhibits sensitivity up to ∼6°C^–1^ (Fig. 2d, Fig. S9b), which is far higher than that of ionogel-based or thermal resistor devices [46,47]. The temperature sensor is insensitive to applied pressure (Fig. 2d, Fig. S10a). We calibrate the pressure response curves with high consistency using the data collected by the temperature sensor (the decoupling method is illustrated in Fig. S10b–d).

Additionally, a strong interfacial bonding strategy is employed for different layers of the bimodal skin to enhance mechanical stability. We show that the sensor remains stable when subjected to a high shear stress of 66 kPa for 2000 cycles (Fig. 2e). We also show that the signal is stable when cycled between 30°C and 40°C for 1000 cycles (Fig. 2f). Furthermore, compared to traditional end-effector six-axis force/torque sensors, our iontronic skin enhances robotic tactile perception over large-area surface, offering improvements in pressure range (1 MPa), pressure resolution (∼0.01 N), number of sensing channels (768 pressure units, 75 temperature units), intrinsic pressure sensing and high-dimensional feature detection (Table S2).

Our circuit allows for rapid and crosstalk-free readout of sensing signals. The readout interface employs the frequency-coding architecture [31] to achieve the high-speed and parallel readout of the addressable matrix sensors with 16-bit resolution (Fig. 2g, Figs S11–S15, Notes S3 and S4). Frequency-encoded excitation signals were applied to different rows during readout (Fig. S13), while the mixed signals were applied to different columns and demodulated via fast Fourier transform in the backend. Our readout circuit facilitates parallel sampling, demodulation, calculation and signal transmission, achieving a readout rate of approximately 93 frames per second (Figs S16 and S17). The crosstalk between sensing units was suppressed via two means: the use of isolated microstructured ionic gel to hinder ion transport from one sensor to another [30]; and the parallel zero-potential mechanism [31], which relies on operational amplifiers and their feedback circuits to maintain zero potential for each column electrode, preventing the electrical crosstalk caused by parallel capacitive coupling between the electrodes. As a result, our sensor array exhibits ∼0.1% crosstalk (Fig. S18), defined as the ratio of the response amplitude of adjacent sensors to that of the target sensor under test.

We further validated the real-time tactile perception of bimodal skin in human–robot interactions. When a hand holds bimodal skin, the patterned signals, whether pressure or temperature, well reflect the shape of the touch (Fig. 2h). Our iontronic skin can map the static pressure of an object without crosstalk (Fig. S19), and provide real-time response to dynamic pressure from hand touch (Fig. S20, Movie S1) We recorded the time-series signals during hand-slapping interactions with the bimodal skin (Figs S21–S23). These multichannel spatiotemporal tactile data can capture the sensory features of different interactive behaviors.

### Edge-intelligent iontronic skins for a walking collaborative robot

**Figure 3.**
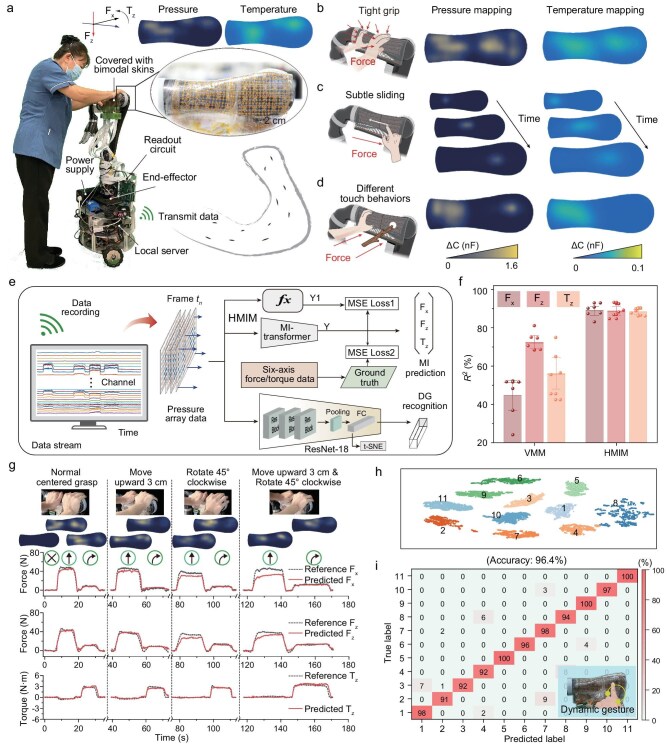
Edge-intelligent iontronic skins for a walking collaborative robot. (a) Photograph of the collaborative robot equipped with bimodal skins. Inset shows the iontronic skins and pressure mapping during hand touching. (b and c) Tactile responses of the large-area coverage iontronic skins to human interaction behaviors, such as gripping and sliding. (d) Tactile responses of the large-area coverage iontronic skins to hand and external object contact. Hand contact triggers a temperature response, while external objects do not. (e) Overall architecture for MI inference and DG recognition. The HMIM framework trains the MI-transformer using a hybrid supervised learning paradigm that integrates both data and knowledge. ResNet-18 is responsible for extracting high-dimensional spatiotemporal tactile features to enable dynamic tactile perception. (f) Comparison between the proposed HMIM and the physics-based VMM model in terms of the average accuracy for MI inference, measured by the goodness-of-fit coefficient *R*^2^. (g) Adaptive measurement of force and torque measured by the bimodal skin for MI inference at different grasping positions (red solid lines). The black dashes are reference force or torque measured using a six-dimensional force/torque sensor. (h) Visualization of the tactile spatiotemporal features extracted by ResNet-18 from the 11 interaction gestures in the DG dataset using t-SNE dimensionality reduction. (i) Confusion matrix for the recognition of the 11 different interaction gestures using the integration of the bimodal skins and ResNet-18. A recognition accuracy of 96.4% is achieved under a cached time-series data frame length of 100. The inset shows a schematic of the interaction with the bimodal skins.

Three bimodal iontronic skin block, comprising a total of 768 pressure sensing units and 75 temperature sensing units, are conformally laminated onto the arm (covering the whole arm) of a walking collaborative robot (Fig. 3a). Additionally, this robot was equipped with a power supply, a readout circuit, and a local server that receives signals for data storage and user behavior inference (Fig. S24). Notably, our iontronic skins can be wrapped around curved surfaces to capture large-scale spatiotemporal tactile information. Compared to a traditional end-effector (six-axis force/torque sensors), our iontronic skins can distinctly differentiate various tactile behaviors based on the pressure and temperature distributions on the large-area surface (Fig. 3b–d, Figs S25 and S26), thereby enabling the acquisition of richer features related to interaction intentions. We demonstrated that when a user touches the skins on the arm, the sensing signals can reflect different modes of motion, such as pushing forward and turning left/right (Movies S2 and S3). Enhancing collaborative robots with flexible surface skins to perceive user interactions and enable real-time collaborative feedback is a promising approach [3,4]. To better adapt to tasks and human partners, collaborative robots must leverage high-throughput tactile information to infer user intention.

Walking collaborative robots aim to achieve intelligent perception and feedback, enabling more effective walking collaboration and companionship with human partners [6,7]. An intent-driven admittance control strategy is employed to dynamically adjust the robot’s linear and angular velocities in response to user-applied forward force and steering torque on the skins [48] (Note S5, Fig. S27), enabling efficient path following. Here, *F*_h_ ∈ [*F_x_,F_z_,T_z_*] represents a set of motion intent (MI) parameters, where *F_x_* and *T_z_* represent the forward force and the torque applied to the walking collaborative robot, respectively, and *F_z_* denotes the vertical supporting force during the assistive interaction. Accurately predicting ${F}_{\mathrm{h}}$ based on high-throughput tactile signals from large-area surfaces and providing feedback in real time presents a challenge. Crosstalk and inference latency in large-scale tactile signals can severely hinder compliant human–robot collaboration. Moreover, utilizing high-throughput tactile signals to understand dynamic touch gestures (DGs) in human–robot interactions is another key function for large-area skins.

Here, we designed a lightweight deep learning framework that enables large-area bimodal skins to interpret human interaction intentions in real time on edge devices, facilitating autonomous decision-making and feedback. We developed the hybrid motion intention model (HMIM) for high-precision, real-time estimation of the user’s MI (${F\,}_{\mathrm{h}}$) during assisted walking, along with a deep residual convolutional network (termed ResNet) capable of accurately recognizing interactive DGs (Fig. 3e). The hybrid supervised learning algorithm of data and knowledge is central to HMIM. By combining the vector mechanics model (VMM; Note S6, Fig. S28) with real assistive data to supervise the training of a lightweight artificial neural network composed of an MI-transformer (Fig. S29), this approach supplements and corrects MI inference errors caused by inaccurate sensor positioning and inconsistent sensor responses. We also employed the residual network as the backbone network to construct a low-complexity ResNet-18 for high-dimensional spatiotemporal tactile feature learning (Fig. S30), enabling real-time and accurate recognition of interactive DGs.

We collected the MI dataset during assisted walking and the DG dataset from touch interactions (Note S7, Fig. S31), and performed 6-fold cross-validation on participants to report the average results. Figure 3f shows the cross-validation results of the goodness-of-fit coefficient *R*^2^ of *F*_h_ (see Fig. S32 for the training of HMIM), where HMIM achieves an accuracy of 88.9% for ${F}_x$ (minimum value: 82.6%; maximum value: 93.7%), 88.8% for ${F}_z$ (minimum value: 83.9%; maximum value: 93.5%) and 88.3% for ${T}_z$ (minimum value: 86.0%; maximum value: 90.7%). Notably, the results demonstrate a significant improvement in the inference accuracy of HMIM compared with that of VMM. VMM, which relies on prior physical knowledge models for solving, introduces prediction errors from factors such as position errors of the sensors, and non-uniformity of the sensing properties of different sensing units in the sensor array. In contrast, HMIM employs data-driven training to further correct data distribution errors, thereby improving the accuracy of MI prediction (Fig. S33, Movie S4). Compared to mainstream deep learning models (Table S3), our MI-transformer achieves high inference accuracy while demonstrating superior computational and storage efficiency [(3.31M floating-point operations (FLOPs) and 3.32M parameters]. It is capable of performing single inference on an edge device (Jetson Xavier NX-16GB) in 3.2 ms. Additionally, we demonstrated that our MI-transformer exhibits strong robustness in MI inference under large-scale tactile perception, accurately inferring even at diverse contact positions with an offset up to 3 cm from the sensor center and a rotation of up to 45° (Fig. 3g). Excessive hand grasp offset reduces the number of activated sensors, consequently diminishing inference accuracy (Fig. S34). Therefore, maintaining a normal human grasp posture during large-area skin interaction is required to ensure optimal performance.

We further trained ResNet-18 with low complexity on the DG dataset to recognize dynamic touch gestures (see Fig. S35a–d for model training and testing). We used t-distributed stochastic neighbor embedding (t-SNE) to visualize the data derived from the output of these feature extraction layers (Fig. 3h), and assessed the effectiveness of our classification algorithm. Points in the same color represent the same gesture category, forming distinct groupings clustered together for the 11 gesture categories. In the cross-validation results, when the time-series data frame length exceeds 100, ResNet-18 achieves an average recognition accuracy of 96.4% on the DG dataset (Fig. 3i, Fig. S35e). ResNet-18 strikes a balance between high recognition accuracy and computational efficiency (90.5M FLOPs and 11.5M parameters) compared with mainstream spatiotemporal feature extraction networks (Fig. S35f). It enables large-scale tactile spatiotemporal feature extraction with relatively low resource consumption, performing a single recognition task in 16.4 ms on an edge device (Jetson Xavier NX-16GB). By pushing deep learning inference closer to the data source, edge intelligence substantially reduces end-to-end latency and communication overhead while enhancing privacy preservation and system robustness [34]. Consequently, for AI-enhanced sensing and control scenarios that are highly sensitive to timeliness, reliability and security, edge intelligence is not merely advantageous but essential (Table S3). The results indicate that our large-area coverage skins, coupled with lightweight deep learning, offer an effective pathway for real-time sensory perception, decision-making and actuation on edge collaborative robots.

### Collaborative actuation enabled by iontronic skins with edge intelligence

**Figure 4.**
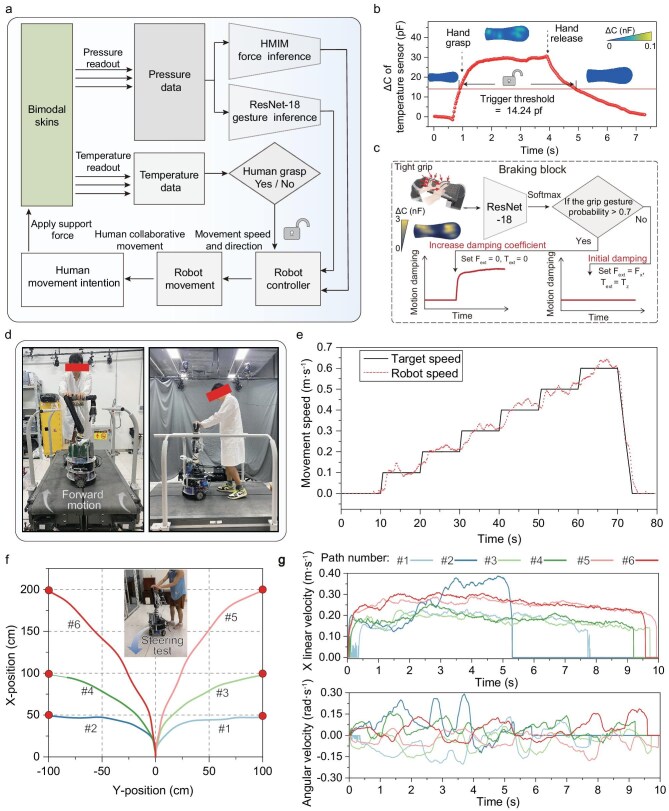
Collaborative actuation enabled by iontronic skins with edge intelligence. (a) System block diagram for the maneuver scheme based on bimodal skins. (b) Plot of the temperature response from the iontronic skins during the process of hand grasp and release (in a ∼22°C indoor environment). The temperature switch is activated when the temperature response exceeds the trigger threshold of 14.24 pF. (c) Flowchart of braking block. When the system detects a gripping gesture, the control module increases damping and sets the desired force and torque to zero to execute emergency braking. (d) Schematic of the collaborative robot performing supportive linear walking on a sliding mechanical platform. (e) The target and true velocities of the robot under velocity increment of 0.1 m$\cdot $s^−1^ from 0 to 0.6 m$\cdot $s^−1^. (f) Steering tests with varying turning radii. Robot motion control is conducted using surface tactile information. The red markers represent the target points reached by the robot, while #1–6 trace the actual movement paths. The illustration depicts the steering process under user tactile interaction. (g) Control responses of the linear and angular velocities of the walking collaborative robot recorded along steering paths #1 to #6.

In the intent-driven walking collaboration paradigm, the walking collaborative robot must perceive user behavior, make decisions and provide real-time feedback to offer compliant walking support and companionship [3,7]. Figure 4a illustrates the maneuvering scheme of the walking collaborative robot utilizing our iontronic skins powered by edge intelligence. Based on user intent reflected in applied pressure and torque, the admittance control system adjusts the robot’s linear and angular velocities [7,48], thereby synchronizing its motion with the user’s gait (Fig. S36). The system is described by Eq. 1 (Note S5):


(1) \begin{eqnarray*}
\left[ {\begin{array}{@{}*{2}{c}@{}}
{{M}_x}&0\\
0&{{M}_\theta } \end{array}} \right]
\left[ {\begin{array}{@{}*{1}{c}@{}}
{\mathop x\limits^{{..}} }\\
{\mathop \theta \limits^{{..}} } \end{array}} \right]
= \left[ {\begin{array}{@{}*{1}{c}@{}} {{F}_{ext}}\\
{{T}_{ext}} \end{array}} \right]
- \left[ {\begin{array}{@{}*{2}{c}@{}}
{{D}_x}&0\\
0&{{D}_\theta }
\end{array}} \right]
\left[ {\begin{array}{@{}*{1}{c}@{}}
{\mathop x\limits^{{.}} }\\
{\mathop \theta \limits^{{.}} } \end{array}} \right],
\end{eqnarray*}

where *M_x_* and *D_x_* are the desired inertia and damping coefficients in the forward displacement *x*, respectively, *M_θ_* and *D_θ_* are the corresponding coefficients for the steering angle *θ, F_ext_* and *T_ext_* represent the expected force and steering torque in the forward direction applied by the user, respectively. The linear acceleration *ẍ* and angular acceleration $\mathop \theta \limits^{{..}} $ are solved by:


(2) \begin{eqnarray*}
\mathop x\limits^{{..}} = \frac{{{F}_{ext} - {D}_x\mathop x\limits^{{.}} }}{{{M}_x}},\
\end{eqnarray*}

(3) \begin{eqnarray*}
\mathop \theta \limits^{{..}} = \frac{{{T}_{ext} - {D}_\theta \mathop \theta \limits^{{.}} }}{{{M}_\theta }}.\
\end{eqnarray*}

The linear velocity and angular velocity at the time step *t* + Δ*t* can be calculated iteratively:


(4) \begin{eqnarray*}
\mathop x\limits^{{.}} \left( {t + {\mathrm{\Delta }}t} \right) &=& \mathop x\limits^{{.}} \left( t \right) + \mathop x\limits^{{..}} \left( t \right) \cdot {\mathrm{\Delta }}t\\
&=& \frac{{{F}_{ext}}}{{{M}_x}} + \left( {1 - \frac{{{D}_x}}{{{M}_x}}} \right)\mathop x\limits^{{.}} \left( t \right),\
\end{eqnarray*}

(5) \begin{eqnarray*}
\mathop \theta \limits^{{.}} \left( {t + {\mathrm{\Delta }}t} \right) &=& \mathop \theta \limits^{{.}} \left( t \right) + \mathop \theta \limits^{{..}} \left( t \right) \cdot {\mathrm{\Delta }}t\\
&=& \frac{{{T}_{ext}}}{{{M}_\theta }} + \left( {1 - \frac{{{D}_\theta }}{{{M}_\theta }}} \right)\mathop \theta \limits^{{.}} \left( t \right),\
\end{eqnarray*}

where Δ*t* is the time interval between time steps, and $\mathop x\limits^{{.}} ( t )$ and $\mathop \theta \limits^{{.}} ( t )$ can be derived from the data of the wheel encoders of the robot and the on-board inertial measurement unit (IMU).

Temperature sensing serves as a safety switch to distinguish secure human hand grasp from false force triggers caused by accidental contact or objects. The motion mode is activated only when the interactive force is confirmed to originate from a secure human hand grasp, as verified by skin surface temperature (Fig. 4b, Fig. S37). Additionally, in the case of danger, humans often instinctively tight grip an object to generate featured force and torque, and the information can be used to trigger braking of the robot. Therefore, we incorporated a braking block that triggers emergency braking when the output probability of the ResNet-18 classifier for the gesture of tight grip, computed via the Softmax function in the output layer, exceeds 0.7 (see Fig. 4c). In the tight grip state, the system increases the damping coefficient and sets *F_ext_* and *T_ext_* to reduce the velocity to zero for safety. The system must ensure that the robot transits to the safe movement mode only when the temperature switch is activated and without triggering the emergency braking (see the motion control flow in Fig. S38). In this mode, *F_x_* and *T_z_* applied to the iontronic skins by the user were used as expected force *F_ext_* and torque *T_ext_*, which were then used in the admittance equation to calculate the expected wheel velocity for speed control.

We further implemented iontronic skin-based collaborative walking tests on our walking collaborative robot. Figure 4d and e demonstrates a test of real-time velocity modulation and sustained walking companionship conducted on a treadmill. The robot system can maintain stable velocity control for coordinated forward motion with the user as the treadmill speed increases from 0 to 0.6 m^–1^. We evaluated the steering performance of the robot by testing its performance to reach six target points along six different paths based on MI exerted by the user. We demonstrate that the robot can control its trajectory at varying turning radii (Fig. 4f), and flexibly adjust its linear and angular velocities to achieve precise motion control within the specified trajectory range (Fig. 4g). Our experiments are configured with higher movement damping, restricting the velocities of the robot to a relatively low level to guarantee the safety of the user. Overall, these results indicate that edge-intelligent iontronic skins can empower robots with intent-driven, real-time interaction, facilitating safe and fluid walking companionship and support.

### Demonstration of real-time walking collaborative interaction via edge-intelligent skins

**Figure 5.**
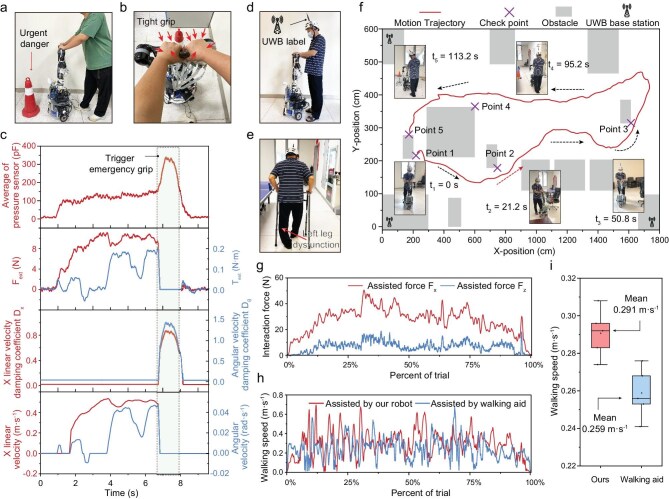
Demonstration of real-time walking collaborative interaction via edge-intelligent skins. (a) Photograph of the collaborative movement in the case of emergency. (b) Tight grip in response to danger. (c) Recorded data of emergency braking when encountering danger during collaborative movement. Upon recognizing a gripping gesture, damping increases and the expected force and torque are set to zero to reduce linear and angular velocities. (d) Photograph of the subject using our walking collaborative robot. The ultra-wideband (UWB) system is utilized to record the subject’s spatial position and movement velocity. (e) Photograph of the subject using a traditional walking aid. (f) The actual walking trajectory of the subject captured by the UWB system, showing the independent mobility process to reach all target points. (g) Tactile interaction forces recorded by our iontronic skins during a single collaborative walking task. (h) Continuous movement velocities using our walking collaborative robot and that of the commercial walking aid during a single walking task. (i) Recorded movement speeds cross five walking tasks performed with our robot and a walking aid. The data validate that our edge-intelligence-driven system can meet the requirements for real-time and compliant collaborative interaction.

We recruited four participants, including three healthy individuals and one male with mobility impairments, to evaluate the controllability and collaborative performance of our robot. Humans often instinctively grip tightly when facing danger, and such a reaction can be captured by the skin system to halt movement for protection. Figure 5a and b simulates the emergency grip braking in response to a sudden emergency during assistive movement. When both hands firmly grip the surface-mounted skins at arbitrary positions, the robotic system detects the key features of the action, a suddenly increased pressure, to trigger the braking block, setting the expected force (*F_ext_*) and torque (*T_ext_*) to zero and increasing damping coefficient for a rapid stop of the robot (Fig. 5c). The results show that the action effectively decelerated linear velocity from ∼0.4 to 0 m$\cdot $s^–1^ in ∼0.1 s.

We aimed to verify that large-scale skins equipped with edge intelligence can enhance collaborative robots on local real-time perception, decision-making and actuation feedback. To this end, we assessed and recorded the real-time collaborative performance of robots during use by the mobility-impaired participant. This subject experienced joint deformity and degenerative changes due to trauma from an external impact on his left leg, resulting in impaired motor function. The condition is characterized by torsional deformity and weakened support, necessitating walking assistance and companionship. While the subject retained some degree of free movement in the left leg, coordinating the assistive device for compliant walking remained challenging. We conducted walking task tests under safety-assured conditions along a designated path (Fig. 5d and e) to verify that our collaborative robotic system is capable of real-time compliant walking collaboration and companionship.

Along the designated path (Fig. S39), the subject achieved coordinated movement with the robot, enabled by collaborative interaction via edge-intelligent skins. We recorded the walking trajectory of the user during the designated task, illustrating that our robot system can achieve collaborative movement to the desired targets through local real-time perception, decision-making and actuation, as illustrated in Fig. 5f and Movie S6. The results indicate that the robot can provide smooth and coordinated mobility collaboration. During the test, the interaction forces along the *x*- and *z*-directions were captured by the large-scale iontronic skins (Fig. 5g). Overall, our robot provided participants with a level of smooth, operable collaboration on par with traditional tools (Fig. 5h and i), with the added benefit of real-time interaction facilitated by large-scale skins with edge intelligence. The limited moving velocity (0.291 m$\cdot $s^–1^) of the collaborative robot is due to safety considerations.

## DISCUSSION

This work introduces a flexible bimodal iontronic skin for large-area robotic coverage, enabling real-time perception of surface pressure and temperature in collaborative robots. By integrating large-scale tactile sensing with lightweight deep learning, the system supports real-time sensory perception, decision-making and actuation within embodied robots. These results suggest that such a skin system can significantly enhance the safety, efficiency and intuitiveness of physical collaboration, particularly in human-centered scenarios. Furthermore, this approach is extendable to broader domains such as humanoid robotics, where full-surface, touch-based perception and interaction are becoming increasingly essential.

## Supplementary Material

nwag111_Supplemental_Files

## Data Availability

All relevant code is publicly accessible at https://github.com/jioy/Bimodal-Skins-Robot.git.

## MATERIALS AND METHODS

Detailed materials and methods are available in the Supplementary data.
