# Supplementary material for: Bimodal iontronic skins powered by edge intelligence for real-time collaborative interaction
Source: Natl Sci Rev. 2026 Feb 14;13(7):nwag111. doi: 10.1093/nsr/nwag111 (PMC13056716; doi:10.1093/nsr/nwag111)
Supplement: nwag111_Supplemental_Files [file nwag111_supplemental_files.zip › Supplementary Materials.pdf]

Supplementary Materials for

Bimodal Iontronic Skins Powered by Edge Intelligence for Real-Time Collaborative Interaction

Zhibin Li[1,†], Junli Shi[2,†], Xinxing Chen[3,4,†], Xingxing Chen[2,†], Ping Yang[4], Hanxiang He[5], Yuquan Leng[4], Xintao Huan[6], Han Hu[5], Chenglong Fu[4,*], Taihong Wang[1,*], Chuan Fei Guo[2,*]

[1]Department of Electronic and Electrical Engineering, Southern University of Science and Technology, Shenzhen 518055, China.

[2]Department of Materials Science and Engineering, Southern University of Science and Technology, Shenzhen 518055, China.

[3]The Key Laboratory of Image Processing and Intelligent Control and the Hubei Key Laboratory of Brain-inspired Intelligent Systems, School of Artificial Intelligence and Automation, Huazhong University of Science and Technology, Wuhan 430074, China.

[4]Department of Mechanical and Energy Engineering, Southern University of Science and Technology, Shenzhen 518055, China.

[5]School of Information and Electronics, Beijing Institute of Technology, Beijing, 100081, China.

[6]School of Cyberspace Science and Technology, Beijing Institute of Technology, Beijing, 100081, China.

†These authors contributed equally to this work.

*To whom correspondence should be addressed. Email: fucl@sustech.edu.cn (C.F.), wangth@sustech.edu.cn (T.W.), guocf@sustech.edu.cn (C.F.G.).

This PDF file includes:

Supplementary Text

Supplementary Figs. 1 to 39

Supplementary Tables 1 to 4

References

Other Supplementary Materials for this manuscript include the following:

Supplementary Movies S1 to S6

MATERIALS AND METHODS

Materials

The following chemicals were purchased from Aladdin, including: 1-Vinylimidazole (V109377), 4-Vinylbenzyl chloride (V592268), benzoin dimethyl ether (BDE, B105178), tri (ethylene glycol) divinyl ether (TDE, T298955), 1,2-bis (2-mercaptoethoxy) ethane (BMOE, D154345), trimethylolpropane tris (3-mercaptopropionate) (TMPT, T464015), 1-ethyl-3 - methylimidazolium bis (trifluoromethylsulfonyl) imide ([EMIM][TFSI], E359386)bis (trifluoromethane) sulfonimide lithium salt (LiTFSI, B102576), mercaptopropyl trimethoxysilane (MPTMS, M100619), 3-(trimethoxysilyl) propyl methacrylate (TMSPMA, S111153), acetic acid (A433221), acetone (A399750), ethylacetate (E116136) and ethanol (E111963). Poly (vinylidene fluoride-co-hexafluoropropylene) (P(VDF-co-HFP), Kynar Flex 2801) was purchased from Arkema. Polydimethylsiloxane (PDMS, Sylgard 184) was purchased from Dow Corning Co., Ltd. All chemicals were used as received without further treatment.

Surface modification of electrodes

Surface modification process was as follows: The surface of electrode was washed in acetone and ethanol in sequence, followed by drying using a nitrogen flow. The solution, which was prepared for surface modification to graft sulfhydryl groups, contained 45 vol.% deionized water, 45 vol.% ethanol, and 10 vol.% MPTMS. The other solution, which was used to graft acrylic double bond groups, contained 45 vol.% deionized water, 45 vol.% ethanol, and 10 vol.% TMSPMA. Before using the two solutions, acetic acid was added dropwise to generate a pH value of 3.0. The electrode was then immersed into the MPTMS solution for 2 min and washed with ethanol and deionized water in sequence for three times, followed by immersing in a TMSPMA solution for 30 min, and washing in ethanol and deionized water for three times.

Synthesis of the ionogel and the PIL

For the synthesis of the ionogel, 0.95 g TMPT, 3.95 g BMOE, 5.10 g TDE, 0.10 g BDE (as the initiator), 2.00 g [EMIM][TFSI], 6.00 g P(VDF-*co*-HFP) (as the thickener) were mixed and with vigorous stirring. The viscous white paste was scratched to the fixed pixel position followed by casting on a microstructured template. Furthermore, the precursor was polymerized via UV light initiation (365 nm) for 3 h for curing. Specifically, 1-vinylimidazole and 4-vinylbenzyl chloride

(1:1 mole ratio) were mixed together followed by stirring at 70℃ for 24 h under N_2 protection. The product was washed three times with ethylacetate to obtain a light yellow viscous ionic liquid (1-vinyl-3-(4-vinylbenzyl) imidazolium chloride ([VVBIM][Cl])) with divinyl groups. For the ion exchange process, the product was dissolved in deionized water. LiTFSI aqueous solution (with a molar ratio between [VVBIM][Cl] and LiTFSI to be 1:3) was added into the solution subsequently, and the mixture was stirred for 24 h for completely reaction. The light-yellow oily precipitate was washed with deionized water three times to form the precursor ([VVBIM] [TFSI]) of PIL. For the synthesis of PIL, the uniform precursor solution containing 2.91 g [VVBIM][TFSI], 0.88 g BMOE, 0.21 g TMPT and 0.04 g BDE was cured via UV light initiation (365 nm) for 3 h.

Characterization of mechanical and electrical properties of the sensors

The force and displacement data for the stress-strain curves in a compression test and that in a peeling test were collected using a force gauge installed on a computer-controlled stage (XLD-20E, Jingkong Mechanical Testing Co., Ltd.). The loading rate for the compression test and peeling test were carried out at a constant rate of 6 mm·min^{-1} and 50 mm·min^{-1}, respectively. The electrical conductivity of PIL was tested using a broadband dielectric impedance spectrometer (Concept 50, Novocontrol Technologies GmbH & Co.KG) at an amplitude of 1 V and in the frequency range from 0.1 Hz to 10 MHz.

Fabrication of the bimodal iontronic skins

The pressure sensor adopts a trilayer configuration, in which a microstructured ionogel is sandwiched between two electrodes. The temperature sensor consisted of a layer of plug-finger electrode and underlying PIL. Both sensors were encapsulated using PDMS membranes and the sensing units were connected by serpentine PI-Cu wires. Details for the fabrication of bimodal iontronic skins can be seen in Note 2 of **Supplementary Text** and **Fig. S6**.

Sensing properties of the bimodal iontronic skins

An LCR meter (E4980AL, KEYSIGHT) was used to measure the capacitance of the sensors. Capacitive measurements were all performed at 100 mV and 1 kHz unless otherwise specified. The pressure applied to the sensors was controlled and recorded by a tensiometer machine (LD23.501, LSD). A lock-in amplifier (MFLI, Zurich Instruments) was used for aliasing signal

acquisition and spectrum analysis. The temperature variation applied to the temperature sensor was controlled and recorded by hot plate (TP102+, Shanghai Hengshang Precision Instruments co., Ltd.).

Development of interface circuitry for sensor signal readout

The 32-bit microcontroller (MCU) STM32H723VGH6 (STMicroelectronics) with a clock speed of 550 MHz was used for the functional control, signal demodulation, and USB transmission. The core operations involved performing fast Fourier transform on mixed frequency signals to demodulate and calculate capacitive responses at different excitation frequencies. The direct digital frequency synthesizer (AD9833, Analog Devices Instruments) generated AC frequency encoded excitation signals. The backend components included an operational amplifier and a high-pass filter, with an output voltage amplitude of 200 mV. The C/V converter uses the precision operational amplifier OPA4388 (Texas Instruments). A 16-bit, 8-channel analog-to-digital converter (AD7606C-16, Analog Devices) was used in the analog signal chain. It operated at a conversion rate of 200 kSPS to digitize voltage signals for demodulation calculations. The analog switch matrix (CH446Q, WCH) handled channel switching within the temperature array.

Construction of the real-time sensory system with a visual user interface

Interactive user software was developed on a computer using Python 3.9 with the PyQt5, PyQtGraph, and OpenCV frameworks. The software supported real-time data transmission via USB or WIFI, enabling waveform visualization, graphical rendering, pressure and temperature map visualization, and data storage.

Design and assembly of collaborative robot

The walking collaborative robot consisted of a supporting robotic arm (KINOVA GEN2, Kinova Robotics) and a mobile base with an omnidirectional wheel drive system. It was equipped with a power supply, a six-axis force/torque sensor (M3564F, Sunrise Instruments) as the end-effector, and a local server (Jetson Xavier NX-16GB, NVIDIA). The server locally managed data reception, wireless communication, model inference, and motion control at the edge. Our robotic control system was based on ROS (Robot Operating System) and developed using Python 3.7 with packages of Rospy and PyTorch to realize functions of data reception, storage, processing, model

inference, node command communication, and hardware scheduling control. Ultra-wideband (UWB) was used for spatial positioning in assistive mobility. Base stations were set up in three corners of the room, and a tag was installed on the safety helmet of user to record the movement process.

Construction of dataset

The MI dataset included distributed tactile data captured by bimodal iontronic skins during simulations along various motion paths, along with reference data collected from six-axis force/torque sensors recorded at 40 Hz with over 200,000 frames. The data were collected from 12 participants of different ages and movement habits (20–54 years old, 8 men and 4 women), with each participant being tested for four rounds (**Fig. S31a**). In each round, the robot remained stationary under high damping, and participants applied the intended control force to the robot according to the guidance of the motion path. The data from the electronic skin was synchronized and collected alongside the real-time data from the ground truth six-axis force/torque sensors. The MI dataset was used to learn and infer the expected forces and torques transmitted by the user through interaction with iontronic skins. The DG dataset included distributed pressure data captured by iontronic skins from 12 participants performing 11 different dynamic touch gestures recorded at 40 Hz for over 50,000 frames, with each gesture repeated for four times. Detailed descriptions of the touch gestures can be found in **Fig. S31b**. The pressure and touch positions reflected individual habits the participants. Cross-validation was conducted among the participants, with data from two users selected as the test dataset, and 6-fold cross-validation was performed across all users, with the average results being reported.

Training and optimization of the network model

A transformer-based HMIM neural network was developed to map distributed pressure values from bimodal iontronic skins to motion intent $\boldsymbol{F}_{\mathrm{h}}$. Being trained with mixed supervision using the Adam optimizer (initial learning rate 0.0001) and MSELoss, the model ran for 10 epochs with a batch size of 40. Additionally, a Resnet network was used to classify dynamic touch gestures from 100-frame tactile data, trained with Adam (learning rate 0.001) and CrossEntropyLoss over 10 epochs. Both models were implemented in PyTorch (Python 3.9) and trained on four NVIDIA T4 tensor core GPUs. The final deployment was on a local server (Jetson Xavier NX-16GB, NVIDIA).

Supplementary Text

Supplementary Note 1. Adhesion mechanism and characterization of sensor interfaces.

As illustrated in **Supplementary Fig. 5(a)**, the schematic representation of the interfaces within two types of sensors is provided. The sensors' structure consists of four distinct interfaces, labeled as Interface I (PDMS|PDMS), Interface II (PDMS|Polyimide (PI)), Interface III (Cu|Ionogel), and Interface IV (Cu|PIL).

Adhesion Mechanism

The adhesion mechanisms of these interfaces are depicted in **Supplementary Fig. 5(b)**.

- Interfaces I and II: Surface hydroxylation of PDMS and PI is achieved via air plasma treatment. Upon contact, hydroxylated PDMS surfaces, as well as PDMS and PI, form Si-O-Si (Interface I) and C-O-Si (Interface II) covalent bonds, respectively, facilitating strong interfacial adhesion.
- Interfaces III and IV: Copper surfaces are modified using silane chemistry. Specifically, copper reacts with thiol groups from (3-Mercaptopropyl) trimethoxysilane (MPTMS) to form Cu–S bonds. The Si-O-Si bonds are formed via hydrolysis reaction between the remaining siloxane groups of MPTMS and siloxane groups of 3-(trimethoxysilyl) propyl methacrylate (TMSPMA), anchoring acrylic ester moieties onto the copper surface. This functionalization enables covalent bonding between PIL/ionogel and copper via a photo-initiated click reaction, ensuring robust adhesion.

Characterization of Interfacial Properties

As illustrated in **Supplementary Fig. 5(c)**, the interfacial toughness Γ(J·m^{-2})) is quantified using a 180° peeling test. The toughness is determined according to Equation (1-1).

$$\Gamma = \frac{F \cdot (1 - cos\theta)}{d} \qquad (1-1)$$

The interfacial toughness Γ is determined using a 180° peeling test, where:

- F represents the force at which the peeling process reaches a steady state.
- d denotes the width of the sample.
- θ is the peeling angle, which is 180° in this case.

As shown in **Supplementary Fig. 5(d)**, the peeling curve of Interface III is presented as an example. The blue curve corresponds to the unmodified interface, serving as the control group, while the red curve represents the peeling behavior of the modified sample. Additionally, **Supplementary Fig. 5(e)** compares the interfacial toughness between the control group and the modified sample. The results indicate a significant increase in toughness following surface modification. A stable interface is essential for ensuring the long-term durability of the electronic skin, particularly under extreme conditions such as loaded under high shear forces.

Supplementary Note 2. Fabrication process of bimodal iontronic skin.

The production process of bimodal iontronic skin is illustrated in **Supplementary Fig. 6**. The fabrication process consists of three primary stages: (1) fabrication of the electrode layer, (2) fabrication of the temperature sensors, and (3) fabrication of the pressuresensors.

(1) Fabrication of the Electrode Layer

The process begins with exposing the surface-modified PI side, followed by spin-coating a PDMS precursor onto its surface. The component is then cured at 80 °C for 2 hours to ensure complete polymerization of PDMS. Subsequently, the copper layer is exposed upwards, and the electrode pattern is defined using laser cutting. After removing excess materials, the pattern is transferred from the glass substrate using water transfer tape. At this stage, the PDMS side becomes exposed upwards. The tape and a separate PDMS film are then plasma-treated simultaneously before being pressed together to achieve adhesion. Finally, the stretchable electrode layer is obtained by removing the water transfer tape via water washing.

(2) Fabrication of the Temperature Sensors

The electrode layer designated for temperature sensing is first immersed in a solution for surface modification, followed by sequential washing with ethanol and deionized water. The treated electrode layer is then placed flat on a glass substrat, and PIL precursor is squeezed at the pixel positions via 3D printer and subsequently cured under UV light. Finally, the electrode layer with solidified PIL and a pre-cured PDMS film used for encapsulation undergo simultaneous plasma treatment, and then pressed together for adhesion, completing the temperature sensors fabrication.

(3) Fabrication of the Pressure-Sensitive Skin

The surface modification process follows the same steps as described above. A polyethylene terephthalate (PET) mask with through-holes aligned to the pixel positions is placed on the bottom electrode layer. The ionogel precursor is then deposited onto the electrode surface using a scraper, ensuring that the precursor infuses into the through-holes. Next, a template is placed over the ionogel precursor to build microstructure on iongel surface, and the polymerization of ionogel is initiated using UV light. After curing, the template and PET mask are removed. The entire component, along with a PDMS spacer (used for maintaining uniform initial values for pressure sensors) undergo plasma treatment, and immediately pressed together for adhesion. Finally, the patterned top electrode, the bottom electrodes with attached spacer and microstructured-ionogel, are encapsulated using the same adhesion method as mentioned above to complete the fabrication of the pressure sensors.

Assembly of bimodal iontronic skin. The temperature sensors and pressure sensors are subjected to plasma treatment and then pressed together, forming the bimodal iontronic skin.

Supplementary Note 3. Calculation of capacitance measurement under frequency coding architecture.

As shown in **Supplementary Fig. 12**, the output voltage of the capacitance-to-voltage converter (C/V converter) can be expressed as follows[1]:

$$V_j'(t) = \sum_{i=1}^{N} -\frac{R_f}{j\omega_i C_f R_f + 1} \cdot j\omega_i C_i \cdot V_i(t) \qquad (2-1)$$

where $V_j'(t)$ is the output signal of the C/V converter on the column interface number j. ω_i is the angular frequency of the excitation source, encoding excitation row number i. N is the total number of encoding channels. R_f and C_f are the feedback resistance and capacitance. C_i is the capacitance on the different column addresses being measured, which can be expressed by solving the corresponding frequency path voltage amplitude:

$$C_i = \left| -\frac{j\omega_i C_f R_f + 1}{j\omega_i V_i(t) R_f} \right| \cdot V_j^i(t) = \frac{\sqrt{\left(\omega_i C_f R_f\right)^2 + 1}}{\omega_i R_f} \cdot \frac{A_j^i}{A_i} \qquad (2-2)$$

where $A_i = 200$ mv is the amplitude of the input AC excitation voltage signal $V_i(t)$ with angular frequency $\omega_i = 2\pi f_i^{freq}$, and A_j^i is the amplitude of the output AC signal $V_j^i(t)$ with angular frequency ω_i. Wherein, A_j^i can be demodulated and solved according to FFT (see **Supplementary Fig. 13**). A_j^i is the amplitude of a signal with a frequency of f_i^{freq}.

Supplementary Note 4. Development of the data acquisition circuit.

Supplementary Fig. 14 and **Supplementary Fig. 15** illustrate the design block diagram and the implementation of the data acquisition circuit, respectively. The data acquisition board consists of five key modules: (1) control module, (2) excitation signal encoding module, (3) signal demodulation module, (4) power supply module, and (5) communication module.

(1) Control Module

The control module comprises a microcontroller unit (MCU), a crystal oscillator, and a reset circuit. The selected microcontroller, STM32H723VGH6 (STMicroelectronics), operates at a clock frequency of 550 MHz, providing the computational capability required for high-speed data acquisition, demodulation calculations, and data transmission. Its primary function is to perform fast Fourier transform (FFT) on mixed-frequency signals to demodulate and compute the capacitive responses at different excitation frequencies.

(2) Excitation Signal Encoding Module

The Direct Digital Synthesizer (DDS), AD9833 (Analog Devices), generates the alternating current (AC) frequency-encoded excitation signals. The output is processed by a backend operational amplifier and a high-pass filter, yielding a voltage amplitude of 200 mV.

(3) Power Supply Module

The data acquisition circuit is powered by a 5V USB source. Within the power supply module: A Low Dropout Regulator (LDO) ensures a stable conversion of 5V to 3.3V to power the MCU. A DC-DC converter generates -5V to supply the operational amplifier's negative rail.

(4) Signal Demodulation Module

The C/V converter employs the precision operational amplifier OPA4388 (Texas Instruments) to convert capacitance variations into corresponding voltage signals. These signals are digitized using a 16-bit Analog-to-Digital Converter (ADC), AD7606C-16 (Analog Devices), which features 8 channels and operated at a conversion rate of 200 kSPS, ensuring accurate voltage signal acquisition for subsequent demodulation calculations. An analog switch matrix (CH446Q, WCH) enables channel switching within the temperature sensor array.

(5) Communication Module

The acquired data is then transmitted from the microcontroller to a local server or PC via USB or WiFi (facilitated by the RF communication chip EMC3090).

Supplementary Note 5. Control parameters in the apparent dynamic model.

Walking collaborative robots require the ability to provide positive feedback based on perceived tactile information to better adapt to tasks or users[2]. The general motion control algorithm for the walking assistance robot based on passive behavior is implemented by applying imposed apparent dynamics[3,4]. These apparent dynamics can be considered as the desired, where the robot's walking assistance behavior is guided by the user's intentions[5]. The admittance control equation is shown in Equation (1).

$$\boldsymbol{M}_{\mathrm{d}}\ddot{\varphi}_{\mathrm{r}} + \boldsymbol{D}_{\mathrm{d}}\dot{\varphi}_{\mathrm{r}} = \boldsymbol{F}_{\mathrm{m}} \qquad (3-1)$$

where $\boldsymbol{M}_{\mathrm{d}}$ and $\boldsymbol{D}_{\mathrm{d}}$ are the desired inertia and damping matrices, respectively. The state vector of robot, $\varphi_{\mathrm{r}} = [x\ \theta]^T$, includes the displacement x in the forward direction and the turning angle θ, which denotes the deviation of the collaborative robot from its forward direction. The human motion intention is described by $\boldsymbol{F}_{\mathrm{m}}$, representing the applied force F_x in the forward direction and the torque T_z to the walking collaborative robot (see **Supplementary Fig. 27**). The structure of $\boldsymbol{M}_{\mathrm{d}}$, $\boldsymbol{D}_{\mathrm{d}}$ and $\boldsymbol{F}_{\mathrm{m}}$ are given by Equations (3-2) - (3-4), respectively:

$$\boldsymbol{M}_{\mathrm{d}} = \begin{bmatrix} M_x & 0 \\ 0 & M_\theta \end{bmatrix} \qquad (3-2)$$

$$\boldsymbol{D}_{d} = \begin{bmatrix} D_x & 0 \\ 0 & D_\theta \end{bmatrix} \qquad (3-3)$$

$$\boldsymbol{F}_{\mathrm{m}} = [F_x\ T_z]^T \qquad (3-4)$$

where M_x and D_x represent the desired inertia and damping coefficients in the forward displacement x, respectively, and M_θ and D_θ represent the desired inertia and damping coefficients for the steering angle θ, respectively. The vertical force F_z represents the support force during the assistance interaction, which is used to assess the support provided by the collaborative robot to the user. $\boldsymbol{F}_{\mathrm{h}}$ as the critical control information that ensures the desired dynamics during human interaction and assistance with the walking collaborative robot, serving as the prediction target for MI, with $\boldsymbol{F}_{\mathrm{h}} \in [F_x, F_z, T_z]$. Therefore, the precise sensing and inference of $\boldsymbol{F}_{\mathrm{h}}$ by the walking collaborative robot equipped with iontronic skins is of paramount importance.

Supplementary Note 6. Description of the Vector Mechanics Model.

In hybrid motion intention model (HMIM), the vector mechanics model (VMM) is a physical model that encapsulates prior physical knowledge[6]. This model relies on the normal forces measured by each pressure sensor unit embedded in the iontronic skins. Using the rotation matrix $\boldsymbol{R}_{\mathrm{s}}$, these forces are spatially remapped to compute the resultant force, denoted as $\boldsymbol{F}_{\mathrm{h}}$.

$$\boldsymbol{F}_{\mathrm{h}} = \boldsymbol{R}_{s} \cdot \boldsymbol{f}_{\mathrm{s}} \qquad (3-1)$$

where, $\boldsymbol{f}_{s}$ represents the normal pressure measured by each pressure sensor unit on the iontronic skins, $\boldsymbol{f}_{s} \in \mathbb{R}^{768\times1}$. The rotation matrix R_{s} can be obtained from the CAD model of the walking collaborative robot equipped with bimodal skins, where $\boldsymbol{R}_{\mathrm{s}} \in \mathbb{R}^{3\times768}$ (the solving process is shown in **Supplementary Fig. 28**).

The VMM is a form of prior physical knowledge. This model neglects the positional errors of the actual sensor attachment, sensor consistency errors, and mechanical structural errors. It relies on the normal forces measured by each sensor unit on the bimodal skins and uses spatial mechanics decomposition to solve the interaction forces in the x and z directions by spatially remapping them. As shown in **Supplementary Fig. 28**, given the known positions and angles α_i of the attached sensors, the normal force f_i of each sensor is decomposed into the x and z directions:

$$F_{x,i} = f_i \cdot \cos(\alpha_i) \qquad (3-2)$$

$$F_{z,i} = f_i \cdot \sin(\alpha_i) \qquad (3-3)$$

Calculate the resultant force in the x and z directions for the entire set of sensors. The resultant force in the x direction for the left armrest is:

$$F_{x\_left} = \sum_{i=1}^{N/2} F_{x,i} = \sum_{i=1}^{N/2} f_i \cdot \cos(\alpha_i) \qquad (3-4)$$

where $1\sim N/2$ represents the sensing units on the left armrest.

The resultant force in the x direction for the right armrest is:

$$F_{x\_right} = \sum_{i=(\frac{N}{2}+1)}^{N} F_{x,i} = \sum_{i=(\frac{N}{2}+1)}^{N} f_i \cdot \cos(\alpha_i) \qquad (3-5)$$

where $(\frac{N}{2}+1)\sim N$ represents the number of sensing units on the right armrest.

The resultant force in the x direction under overall support is:

$$F_x = F_{x_{left}} + F_{x_{right}} = \sum_{i=1}^{N} f_i \cdot \cos(\alpha_i) \qquad (3-6)$$

The resultant force in the z direction under overall support is:

$$F_{\mathrm{z}} = \sum_{i=1}^{N} f_i \cdot \sin(\alpha_i) \qquad (3-7)$$

The rotational torque under overall support is:

$$T_z = (F_{x_{right}} - F_{x_{left})} \cdot \mathrm{L} = (\sum_{i=\left(\frac{N}{2}+1\right)}^{N} f_i \cdot \cos(\alpha_i) - \sum_{i=1}^{\frac{N}{2}} f_i \cdot \cos(\alpha_i)) \cdot \mathrm{L} \qquad (3-8)$$

L represents the distance between the center points of the left and right bimodal skins and the center of the support robotic arm (see **Supplementary Fig. 28d**).

Based on equations (3-6) to (3-8), the collection of sensor units $\boldsymbol{f}_{\mathrm{s}} \in \mathbb{R}^{768\times1}$, after spatial remapping, can be used to determine $\boldsymbol{F}_{\mathrm{h}} \in [F_{x,} F_{z}, T_{z}]$.

$$\boldsymbol{F}_{\mathrm{h}} = \boldsymbol{R}_{\mathrm{s}} \cdot \boldsymbol{f}_{\mathrm{s}} \tag{3-9}$$

where $\boldsymbol{R}_{\mathrm{s}} \in \mathbb{R}^{3\times768}$ is the rotation matrix coefficient in equations (3-6) to (3-8), obtained from the CAD model of the sensor attachment spatial positions.

Supplementary Note 7. Description of the dataset based on walking collaborative robot.

To develop and train an inference model for human movement intentions (MI) and interactive dynamic gestures (DG) under the distributed pressure mapping of bimodal skins, we collected a large-scale dataset from a walking collaborative robot. This dataset consists of two subsets: **(1)** a MI dataset recorded during assisted walking and **(2)** a DG dataset collected under touch interactions.

(1) MI Dataset: Assisted Walking Interaction

The MI dataset comprises distributed tactile pressure data captured by bimodal skins, along with six-axis force/torque sensor data, recorded at 40 Hz over more than 200,000 frames. Participants simulated intended interactive forces based on various motion path prompts.

- Data was collected from 12 participants of varying ages and movement habits.
- Each participant completed four rounds of data collection (**Supplementary Fig. 31a**).
- During the experiment, the robot remained stationary under high damping to prevent movement.
- Participants were given motion path prompts, guiding them to apply intended control interaction forces to the assistive robot.
- Data from the bimodal skin and end-effector (six-axis force/torque sensor) were recorded for corrective learning.

(2) DG Dataset: Dynamic Touch Interaction

The DG dataset includes tactile data captured by bimodal skins from 12 participants, each performing 11 distinct dynamic touch gestures. The dataset consists of more than 50,000 frames, recorded at 40 Hz, with each gesture repeated four times (**Supplementary Fig. 31b**).

For cross-validation among participants, we selected data from two users as the test dataset and performed 6-fold cross-validation across all users, reporting the average results.

Supplementary Figures

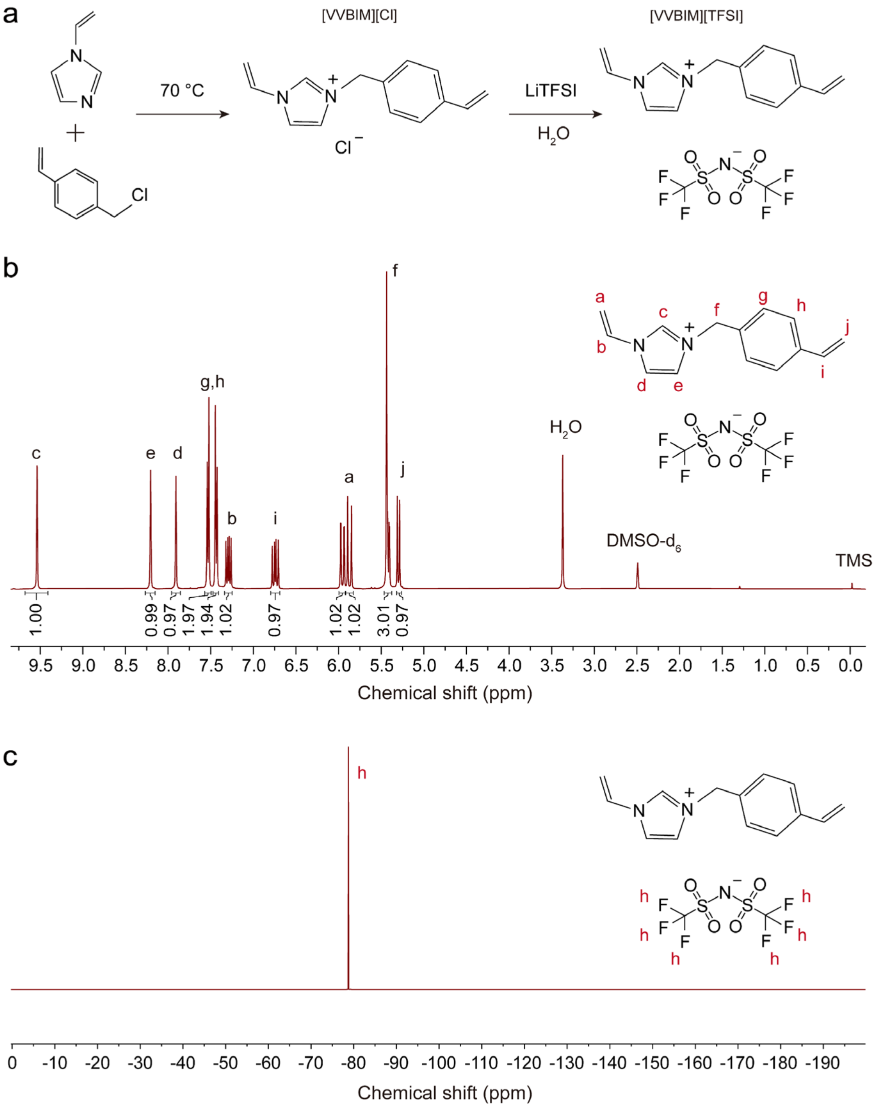

Supplementary Figure 1. Synthesis of diolefin ionic liquid monomer (1-vinyl-3-(4-vinylbenzyl)imidazolium bis(trifluoromethylsulfonyl)imide ([VVBIM][TFSI])). (a) synthetic flow chart of [VVBIM][TFSI]. **(b)** 1H-NMR spectrum (400 MHz, Dimethyl sulfoxide-d, 298K) δ(ppm): 9.53(s,1H), 8.20 (dt, 1H), 7.91(dt,1H),7.53(dd, 2H), 7.43(dd, 2H), 7.29(dd, 1H), 6.74(dd, 1H), 5.95(dd, 1H), 5.87(d, 1H), 5.43(s, 2H), 5.42 (dd, 1H), 5.29 (d, 1H). **(c)** F19-NMR spectrum.

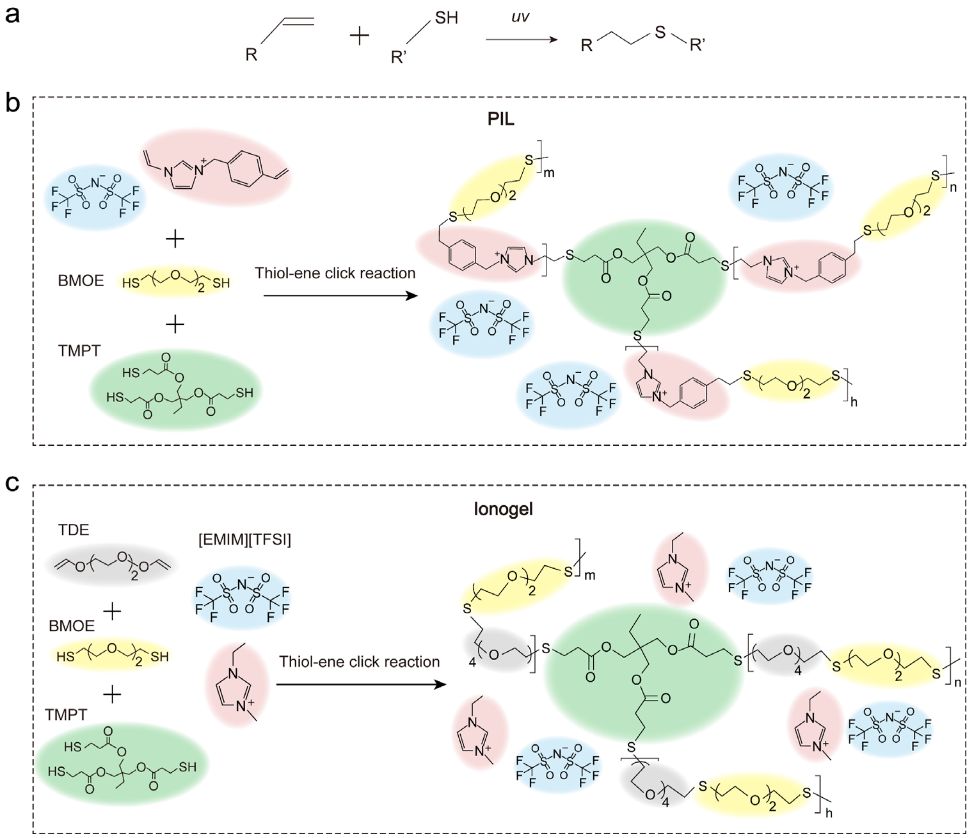

Supplementary Figure 2. Thiol-ene click reaction mechanism and polymerization process of ionic crosslinked polymer utilized for our sensor. (a) Thiol-ene click reaction mechanism. The carbon-sulfur bond is formed by the click reaction between the thiol group and the -ene group from alkene via photoinitiation[7]. **(b)** The reaction mechanism of poly-ionic liquid (PIL). The diolefin ionic liquid monomer ([VVBIM][TFSI], details see **Supplementary Fig. 2**), dithiol monomer (1,2-bis (2-mercaptoethoxy) ethane (BMOE)) and trithiol crosslinker (trimethylolpropane tris (3-mercaptopropionate) (TMPT)) are mixed together with the mole ratio 1:1 between ene and thiol group, and the crosslinked PIL network is formed via photo-initiated click reaction. **(c)** The reaction mechanism of ionogel. Ionic liquid (1-ethyl-3-methylimidazolium bis(trifluoromethylsulfonyl)imide [EMIM][TFSI]), diolefin monomer (tri (ethylene glycol) divinyl ether (TDE)), dithiol monomer (BMOE) and trithiol crosslinker (TMPT) are mixed together with the mole ratio 1:1 between ene and thiol group, and the crosslinked ionogel is formed via photo-initiated click reaction.

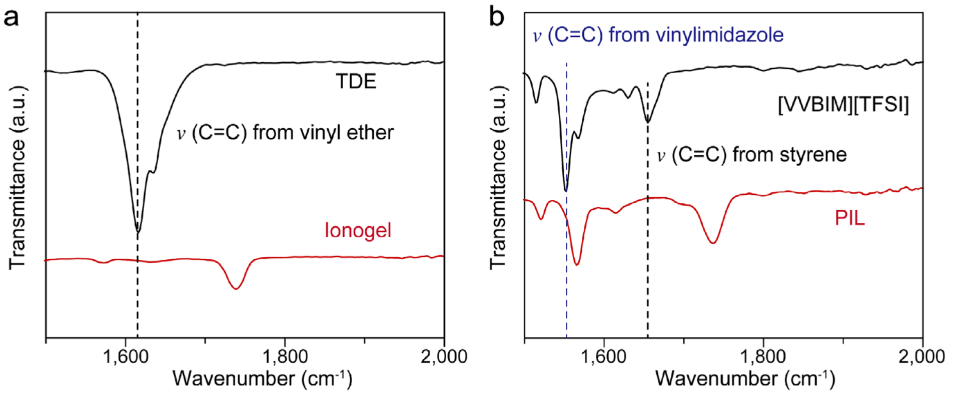

Supplementary Figure 3. Attenuated total reflection infrared (ATR-IR) transmission spectrum of the polymer and precursor. (a) The ATR-IR spectra of TDE and ionogel. It is observed that the fingerprint peak of ν(C=C) from vinyl ether[8] in TDE disappear after click reaction, which indicates that the vinyl group reacted completely. **(b)** The ATR-IR spectra of [VVBIM][TFSI] and PIL. It is observed the fingerprint peak of ν(C=C) from vinylimidazole[9] and styrene[10] disappear after click reaction, which indicates that the vinylimidazole and styrene group reacted completely.

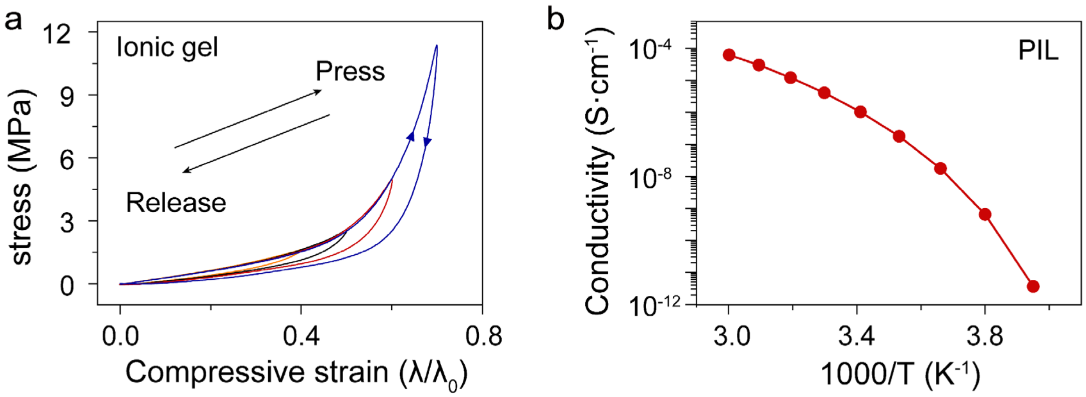

Supplementary Figure 4. Characterization of ionogel and PIL. (a) Press-release curve of ionogel. The ionogel exhibits full recovery under 40%-70% compressive strain, demonstrating notable resilience. This property significantly enhances the recovery performance of the pressure sensor. **(b)** Ionic conductivity of PIL varies with temperature. The curve satisfies VTF equation[11], which demonstrates that the PIL is under high elastic state throughout the tested temperature range. Besides, the conductivity spans two orders of magnitude within the practical application range (10-60 °C), facilitating the development of highly sensitive temperature sensors.

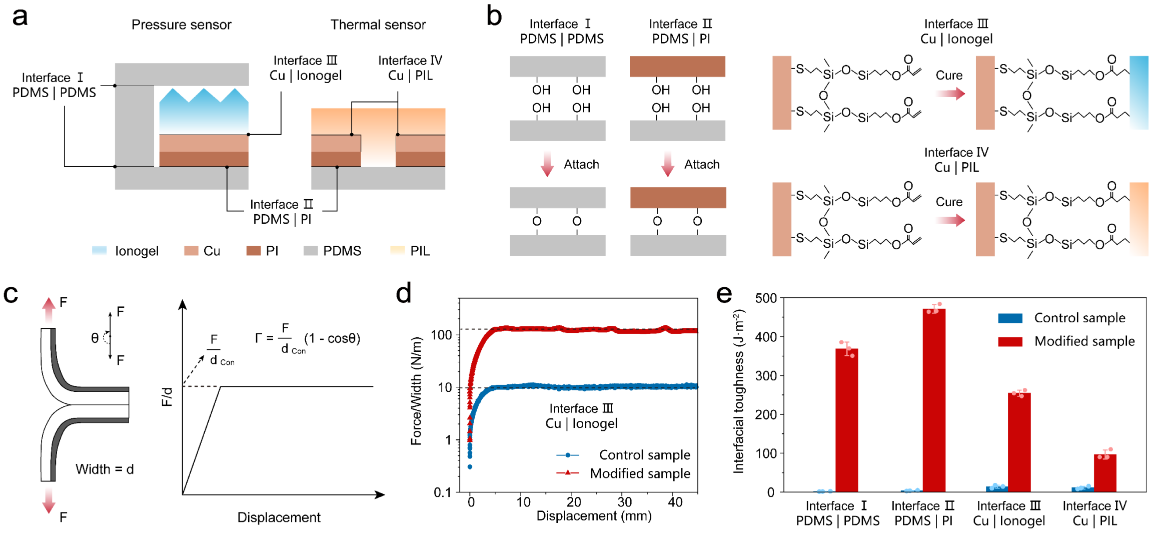

Supplementary Figure 5. The adhesion mechanism and characterization of the interfaces inside sensors. (a) The schematic of the interfaces inside two types sensors. **(b)** Adhesion mechanism of four types interfaces. **(c)** The testing principle of interface property (i.e. interfacial toughness $\boldsymbol{\Gamma}$(J·m^{-2})). **(d)** The peeling curve of interface III is used as an example. The blue one represents the peeling curve of the unmodified interface as control group, while the red curve represents the peeling curve of modified sample. **(e)** Interfacial toughness of control group and the sample after surface modification, while the modified interfaces demonstrate several orders improvement of interfacial toughness compared to control samples.

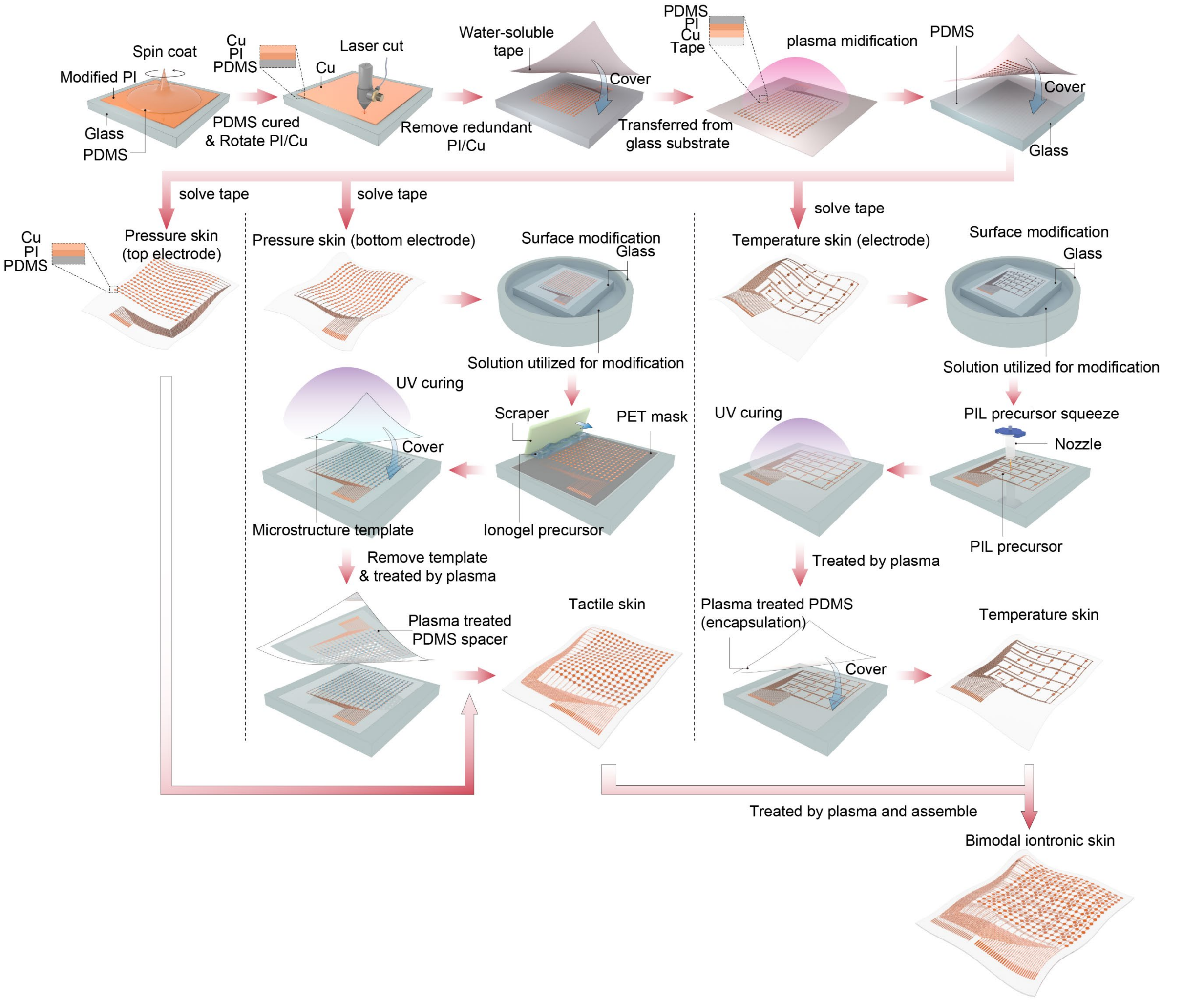

Supplementary Figure 6. The fabrication process of bimodal iontronic skin. The process can be divided into three parts: the fabrication of electrode layer, temperature ssensors, pressure sensors respectively.

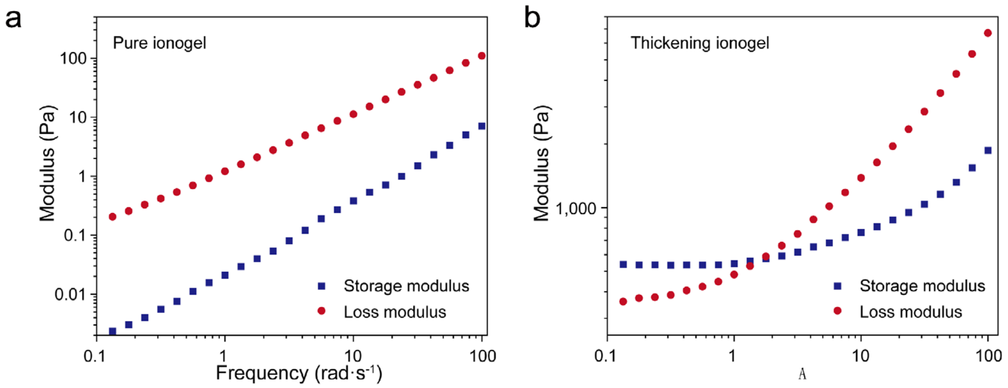

Supplementary Figure 7. Rheological curve of ionogel precursor. We select poly (vinylidenefluoride-*co*-hexafluoropropylene (P(VDF-*co*-HFP)) to thicken the precursor of ionogel. **(a)** The precursor without adding P(VDF-*co*-HFP) remains in a fluid state regardless of whether shear force is applied. **(b)** The thickened precursor demonstrates shear-thinning behavior, which allowing it to be easily scraped like fluid while maintaining adhesion to the designated pixel positions like solid without flowing.

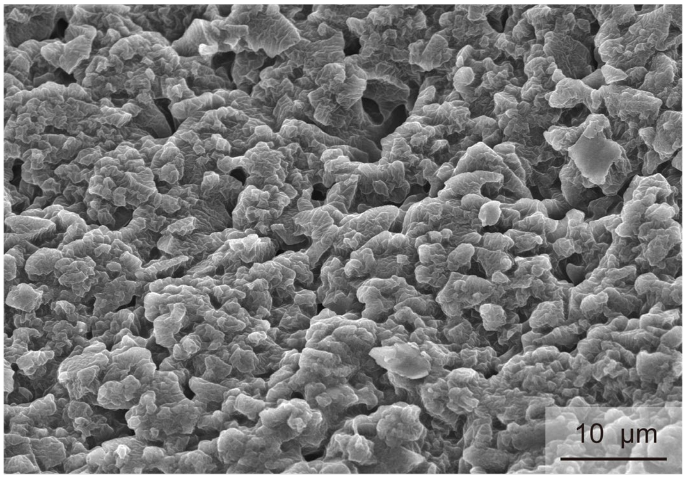

Supplementary Figure 8. Scanning electron microscopy (SEM) image of the microstructure on ionogel surface. A microstructure with a feature size of 5–10 μm can be fabricated on the surface of ionogel using soft lithography, thereby enhancing the sensitivity of the pressure sensor.

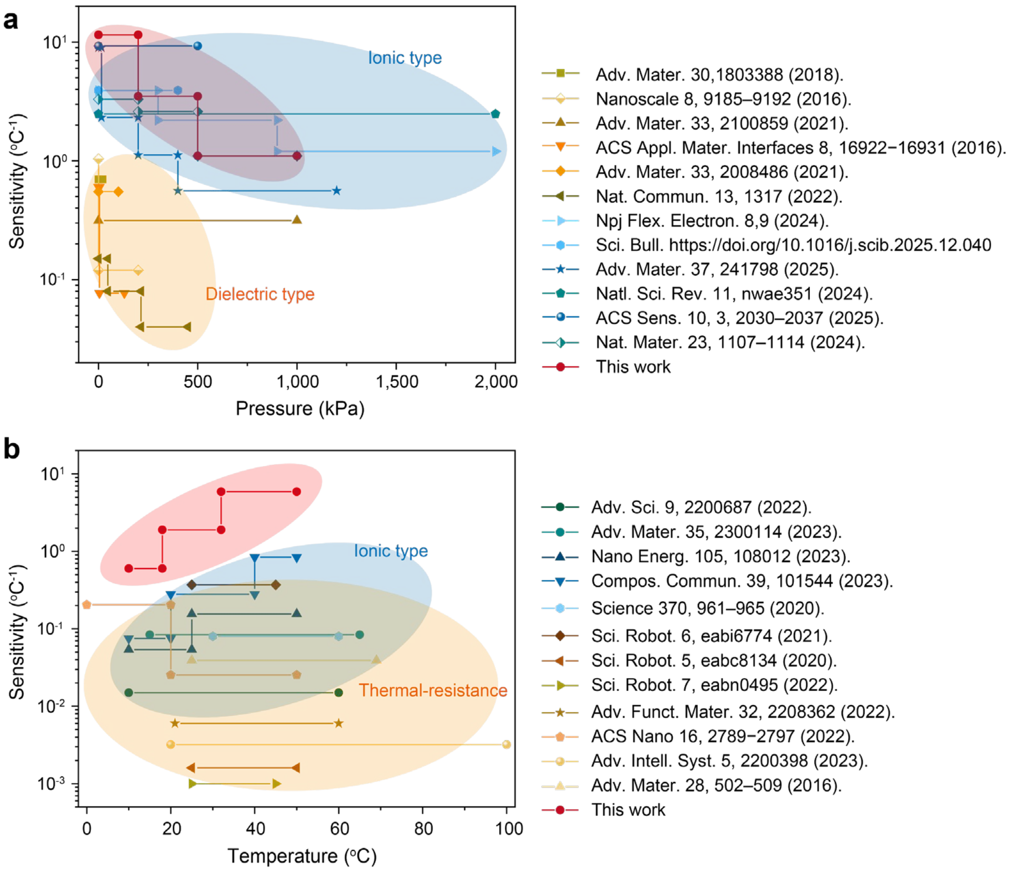

Supplementary Figure 9. Sensitivity comparison of the sensors. (a) Sensitivity comparison between iontronic and dielectric pressure sensors. In iontronic sensors, the capacitance density of the electrical double layer (formed at the interface between ions (ionogel) and electrons (electrodes) at the nanometer scale) is significantly higher than the volumetric capacitance density of dielectric-type sensors. Consequently, our iontronic pressure sensor exhibits superior sensitivity compared to dielectric-type sensors. **(b)** For temperature sensors, ion dissociation and diffusion are more responsive to temperature changes than phonon scattering in metallic materials, resulting in higher sensitivity for ion-based temperature sensors. Additionally, although polymerized ionic liquid (PIL) exhibits lower initial conductivity than ionogel, both materials achieve comparable conductivity at high temperatures. Therefore, PIL-based temperature sensors demonstrate higher sensitivity compared to ionogel-based sensors.

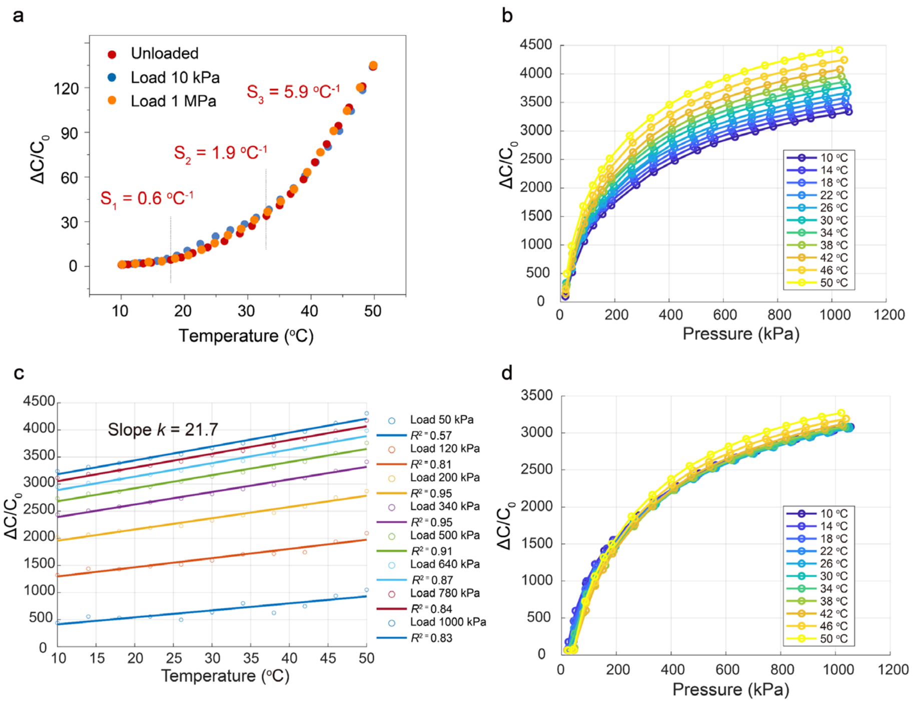

Supplementary Figure 10. The decouple method of bimodal skin. (**a**) *$\Delta C/C_0$-T* curve of the temperature sensor with external pressure of 10 kPa and 1 MPa. The temperature sensor features stress insensitivity. **(b)** *$\Delta C/C0$-P* curves of the pressure sensor under different temperature. It needs to rely on temperature sensor to calibrate the response at different temperatures. **(c)** $\Delta C/C_0$-*T* curves of the pressure sensor under different pressure, used to calibrate temperature-induced variations. To simplify the calculations, the sensor's response with temperature is approximated as linear, with the average slope of the least-squares fit being (k = 21.7), and the fitted result average goodness-of-fit coefficient $\boldsymbol{R^2 = 0.84}$. (**d**) Calibrated $\Delta C/C_0$-*P* curves. The pressure response at different test temperatures is converted to the equivalent response at 10°C. A linear mapping function is used to transform the responses at different test temperatures to their corresponding equivalent values at 10°C, ensuring consistency in sensitivity curve of pressure sensor across different temperatures.

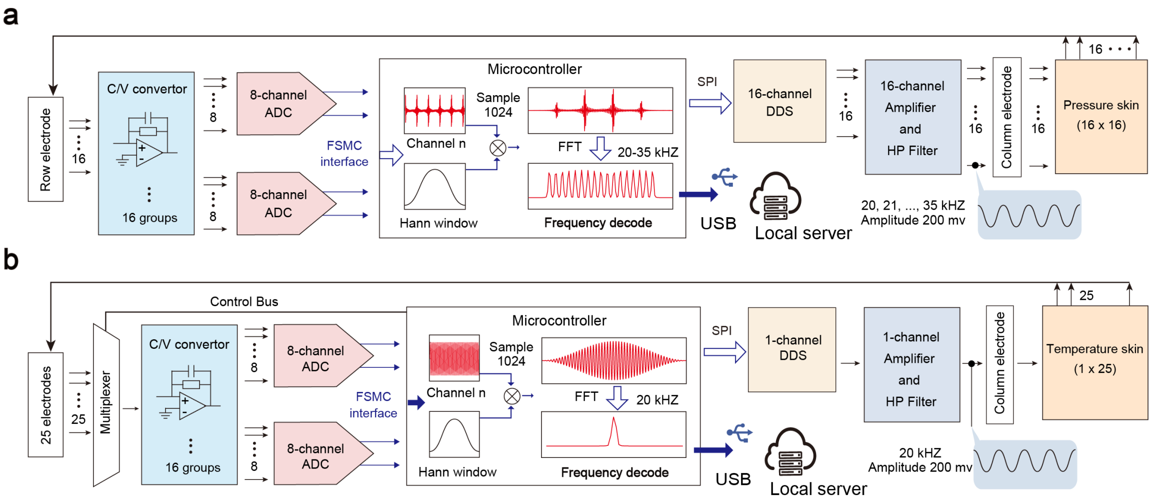

Supplementary Figure 11. Architectural diagram of the bimodal iontronic skin readout interface. (a) Schematic of the 16×16 iontronic pressure array architecture. Sixteen Direct Digital Frequency Synthesizers (AD9833, Analog Devices) are implemented, with backend components including operational amplifiers and high-pass filters to generate excitation signals within the frequency range of 20–35 kHz and a voltage amplitude of 200 mV. Sixteen-channel capacitance-to-voltage (C/V) converters and an analog-to-digital converter (ADC, AD7606-16C, Analog Devices) process the voltage signals from the analog signal chain. The microcontroller then performs fast Fourier transform (FFT) demodulation on the mixed signals. **(b)** Schematic of the 1×25 iontronic temperature array architecture. Unlike the pressure sensor design, a single 20 kHz excitation signal is utilized, with an analog switch incorporated at the receiving end to sequentially acquire voltage signals from 25 channels. Abbreviations: DDS, direct digital synthesizer; OP-AMP, operational amplifier; HP filter, high-pass filter; C/V converter, capacitance-to-voltage converter; ADC, analog-to-digital converter; FSMC, Flexible Static Memory Controller; SPI, Serial Peripheral Interface.

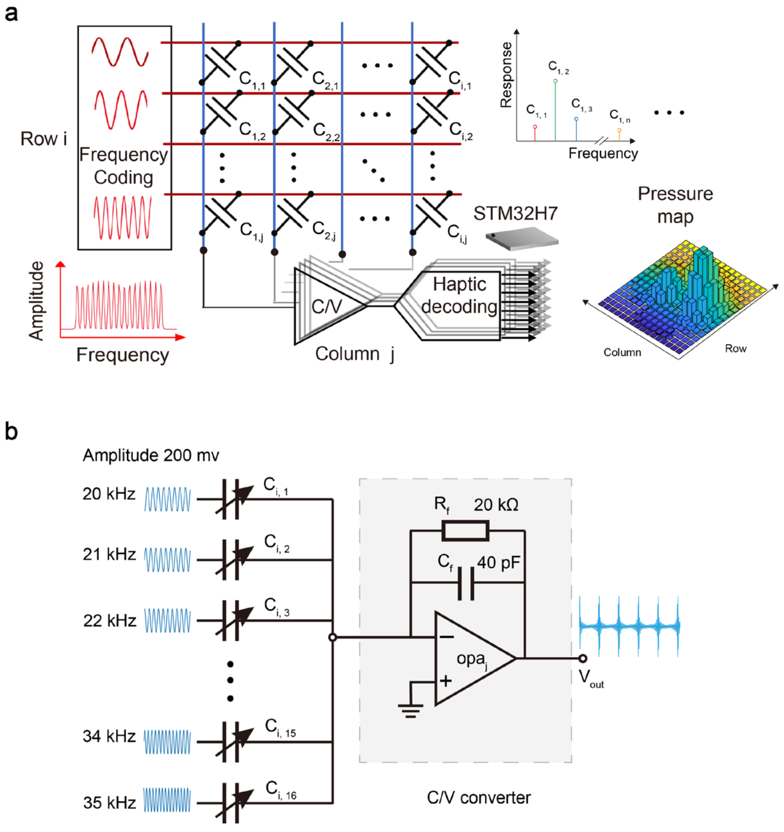

Supplementary Figure 12. Calculation of capacitance measurement under the frequency coding architecture. (a) System diagram of the frequency coding architecture. **(b)** Schematic representation of the parallel summation of capacitive nodes by the capacitance-to-voltage (C/V converter).

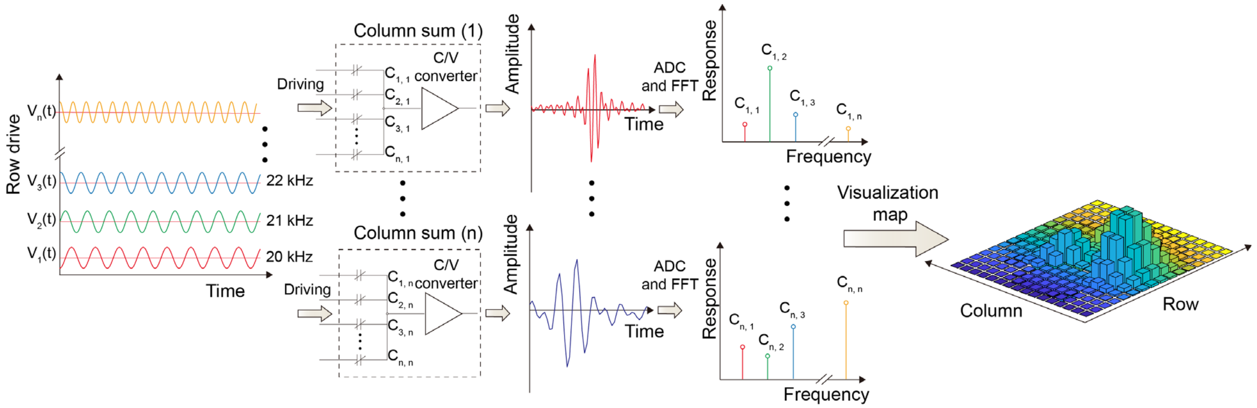

Supplementary Figure 13. Mechanism of the frequency-encoding architecture. Each sensor unit within the matrix is assigned a unique excitation signal encoded with a distinct sinusoidal wave frequency. The capacitance response of each sensor correlates with the amplitude of the corresponding sinusoidal wave. Analog signals from sensor units in each column are aggregated via a capacitance-to-voltage (C/V converter). A real-time fast Fourier transform (FFT) algorithm is then applied to decompose the time-domain composite signal, producing a spectral representation that captures the responses of individual sensor nodes. Finally, the responses of sensor units at different encoding frequencies are reconstructed into a visual map. By discrete the Fourier transform theory, the frequency-bin resolution is expressed as: $\boldsymbol{\Delta f_{bin}} = \frac{\boldsymbol{F_s}}{\boldsymbol{N}} = \frac{\boldsymbol{1}}{\boldsymbol{T_s}}$, where $\boldsymbol{F_s}$ is the sampling rate of the analog-to-digital converter (ADC), $\boldsymbol{N}$ is the number of sampling points, and $\boldsymbol{T_s}$ is the total sampling period. The limitation of frequency encoding is that there must be a sufficient guard interval ($\boldsymbol{f_{inter}} > \boldsymbol{\Delta f_{bin}}$) between carrier frequencies to prevent interference from spectral overlap. Using an ADC with a sampling rate of $\boldsymbol{F_s}$ = 200 kHz and $\boldsymbol{N}$ = 1024 sample points, the required guard interval, based on equation (1-1), is $\boldsymbol{\Delta f_{bin}}$ = 195.3 Hz. In our encoding system, we use excitation signals ranging from 20 kHz to 36 kHz with a 1 kHz interval to measure the 16 × 16 electrode array. This configuration can effectively prevent interference between different frequencies.

1

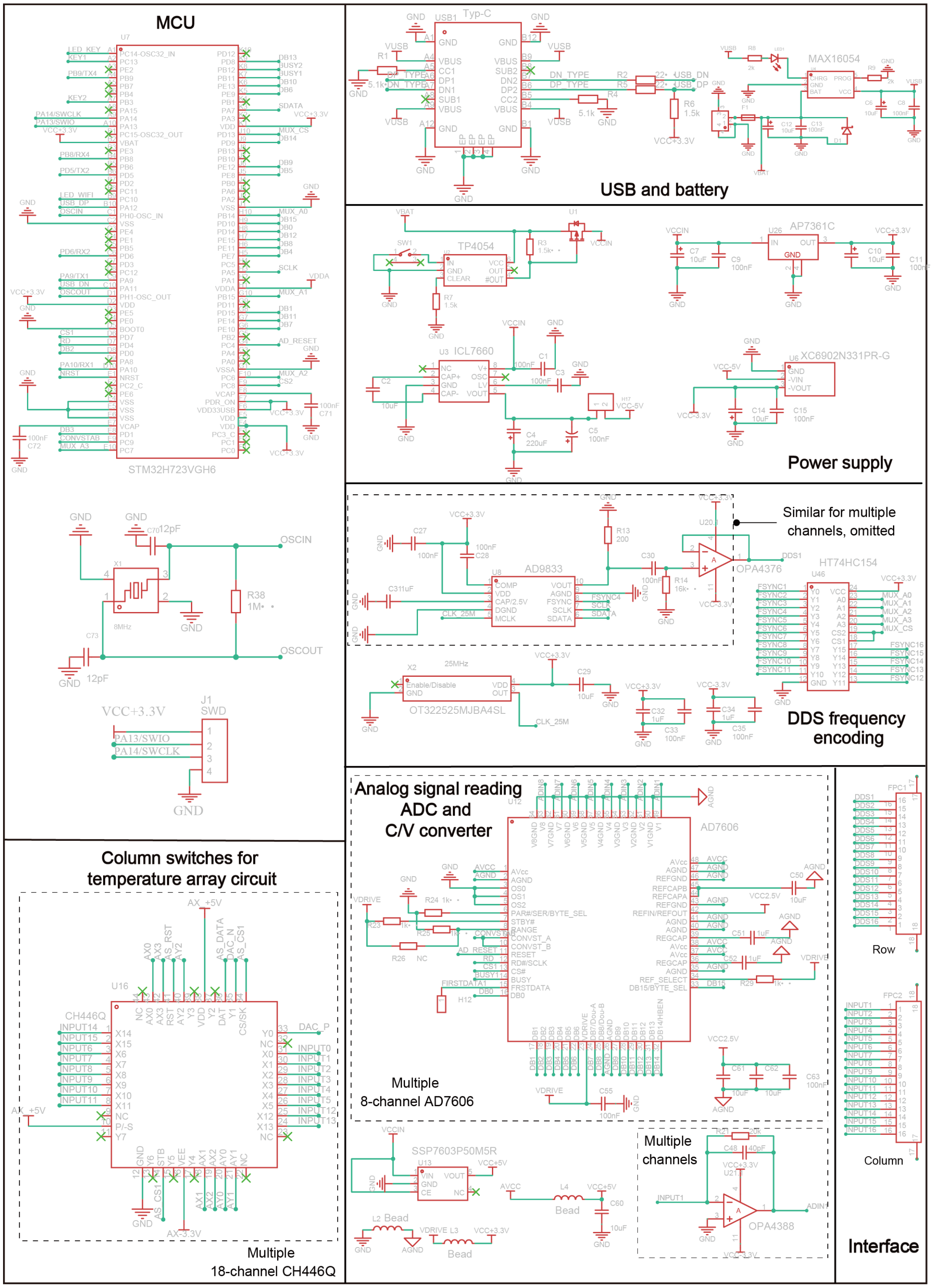
MCU
STM32H723VGH6
USB and battery
MAX16054
Power supply
TP4054
AP7361C
ICL7660
XC6902N331PR-G
Similar for multiple channels, omitted
AD9833
OPA4376
HT74HC154
OT322525MJBA4SL
DDS frequency encoding
Analog signal reading
ADC and
C/V converter
AD7606
Multiple
8-channel AD7606
SSP7603P50M5R
Multiple channels
OPA4388
Column switches for
temperature array circuit
CH446Q
Multiple
18-channel CH446Q
Row
Column
Interface

Supplementary Figure 14. Schematic Diagram of Core Components in the Data Acquisition Circuit. This diagram presents an overview of the key modules and components, highlighting the essential functional units of the microcontroller unit (MCU), USB interface, power supply, frequency encoding, analog signal processing and acquisition, analog switch units, and sensor connection interfaces.

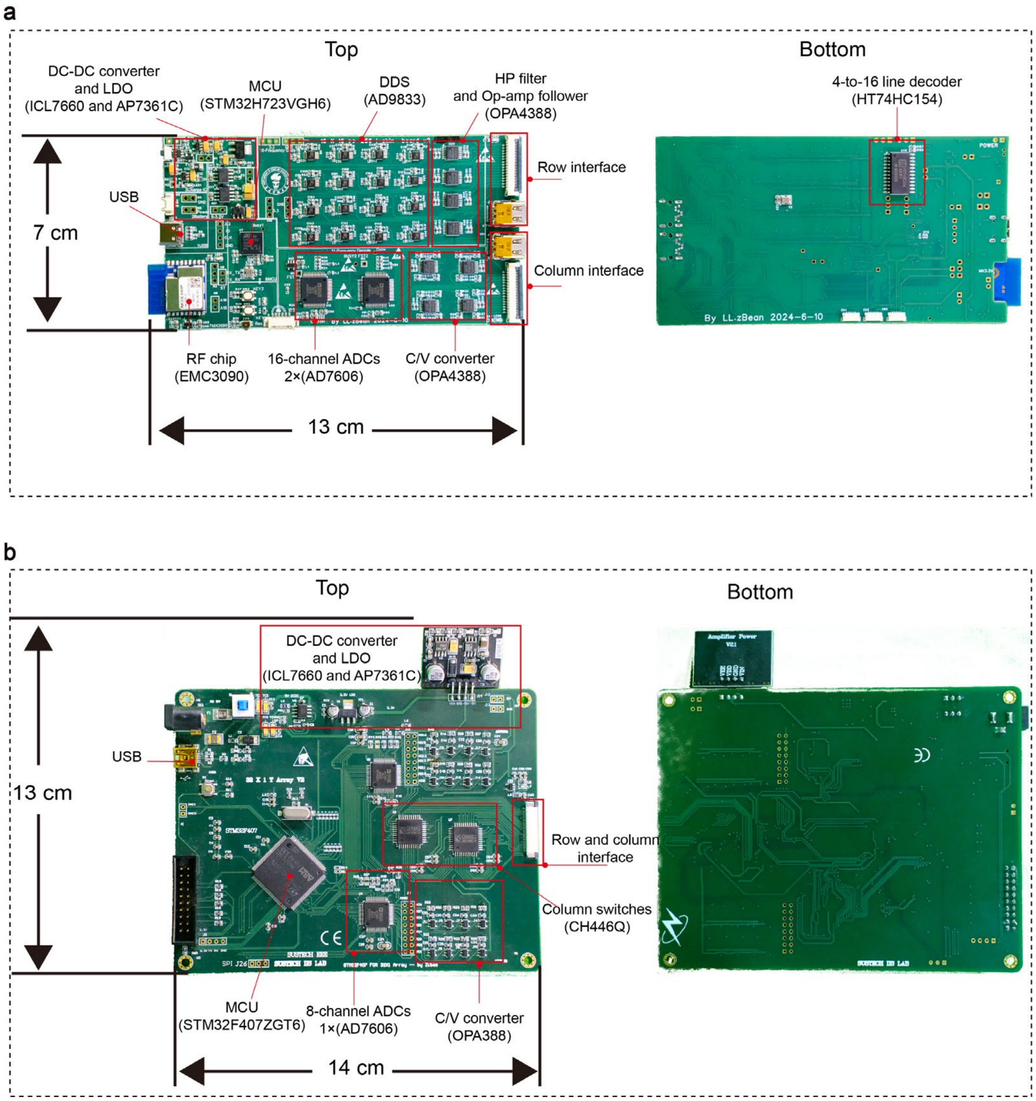

Supplementary Figure 15. Physical PCB layout of the data acquisition board. (a) Printed circuit board (PCB) layout for the pressure skin array acquisition circuit. **(b)** PCB layout for the temperature skin array acquisition circuit. All chip models and component types are labeled in the diagram.

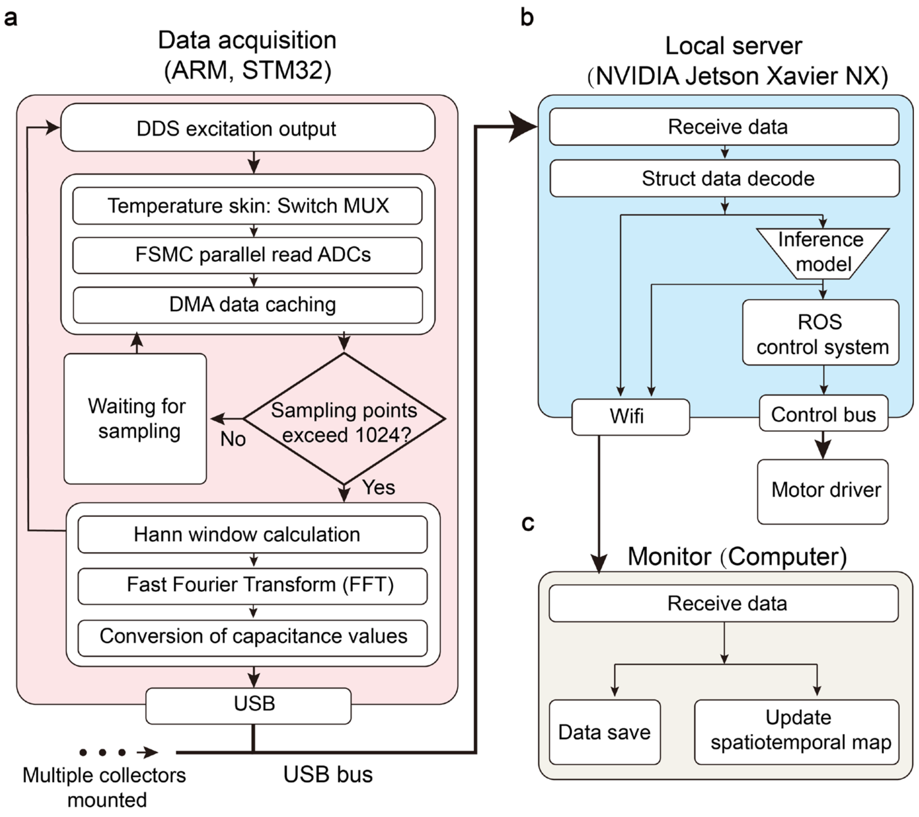

Supplementary Figure 16. Schematic diagram illustrating the principle and operation of the data acquisition system. (a) Working principle of a data acquisition module. **(b)** Local server responsible for data collection and parsing. **(c)** User monitor for data storage and visualization.

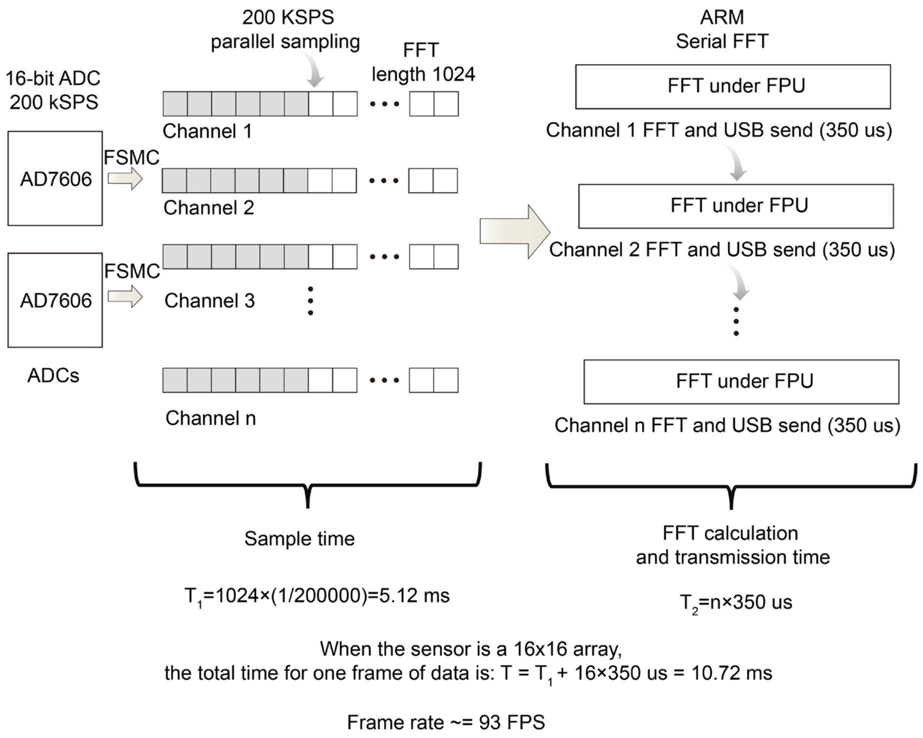

Supplementary Figure 17. Description of the readout circuit frame rate measurement. The total time expenditure of the measurement circuit is primarily composed of data sampling time and the time required for fast Fourier transform (FFT) computation and data transmission. Abbreviations: FPU, Floating Point Unit; FPS, Frames Per Second.

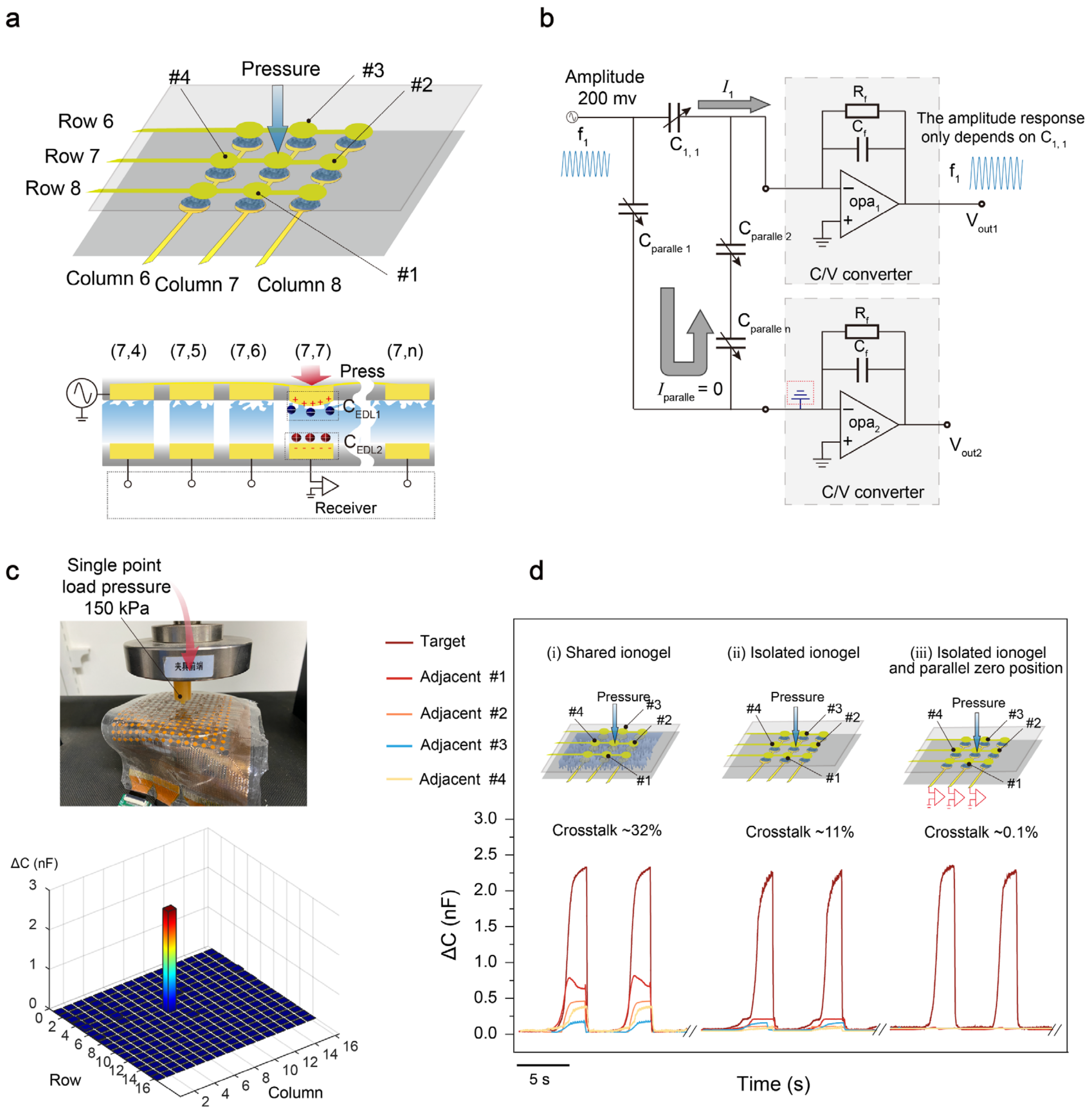

Supplementary Figure 18. Crosstalk suppression analysis in iontronic array. (a) Structure diagram of the isolated microstructured ionic gel design**.** Each sensor unit has its own independent ion gel, effectively preventing ion migration crosstalk between units [12]. **(b)** Schematic of electrical crosstalk in parallel circuits of the array**.** The parallel zero-potential mechanism prevents current conduction from other parallel capacitors, effectively isolating electrical crosstalk [1]. **(c)** Array signal response map under single-point load of 150 kPa pressure. **(d)** Crosstalk response test under a single-point load cycle pressure of 150 kPa. Crosstalk is defined as the ratio of the response amplitude of adjacent sensors to that of the target sensor under test. $Crosstalk = (\Delta C_{adjacent})/(\Delta C_{target})$, ΔC_{target} represents the capacitance change of the target sensor under pressure, while $\Delta C_{adjacent}$ represents the capacitance change induced by crosstalk from adjacent sensors. When segmented ion gel membranes are used, the crosstalk is reduced from 32% to 11%. Additionally, with the suppression of electrical crosstalk through the parallel zero-potential mechanism, the crosstalk is further reduced from 11% to 0.1%.

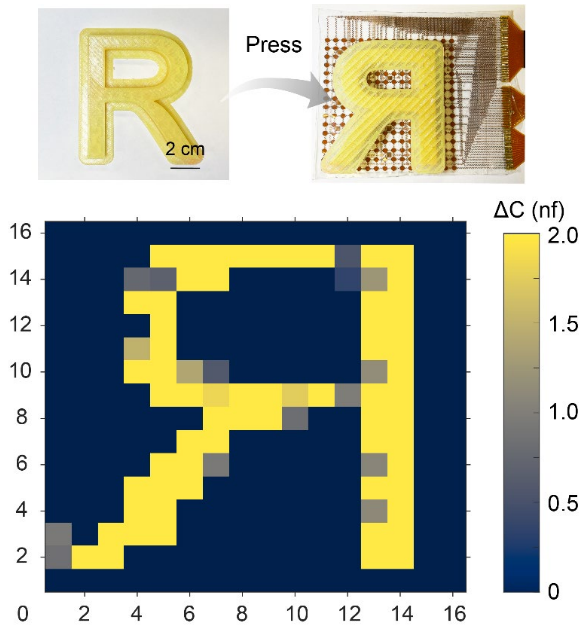

Supplementary Figure 19. The pressure signal mapping of the iontronic skin is obtained under a uniform static pressure of approximately 4 kPa, which can display the shape of a 3D-printed object with crosstalk-free accuracy.

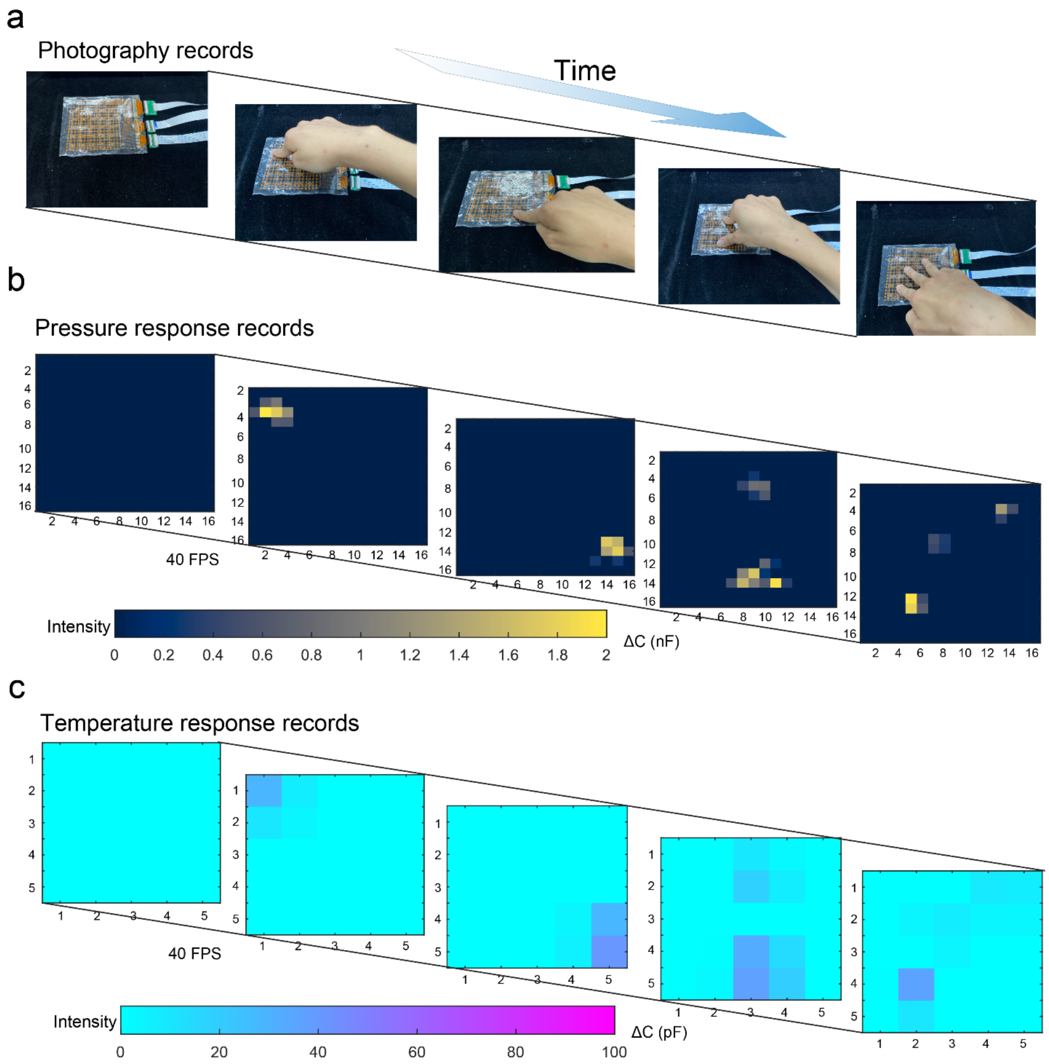

Supplementary Figure 20. Bimodal skin response under dynamic stimuli for different actions. **(a)** Photographs of dynamic stimuli. **(b)** Dynamic pressure signal mapping during the stimulus process. **(c)** Dynamic temperature signal mapping during the stimulus process.

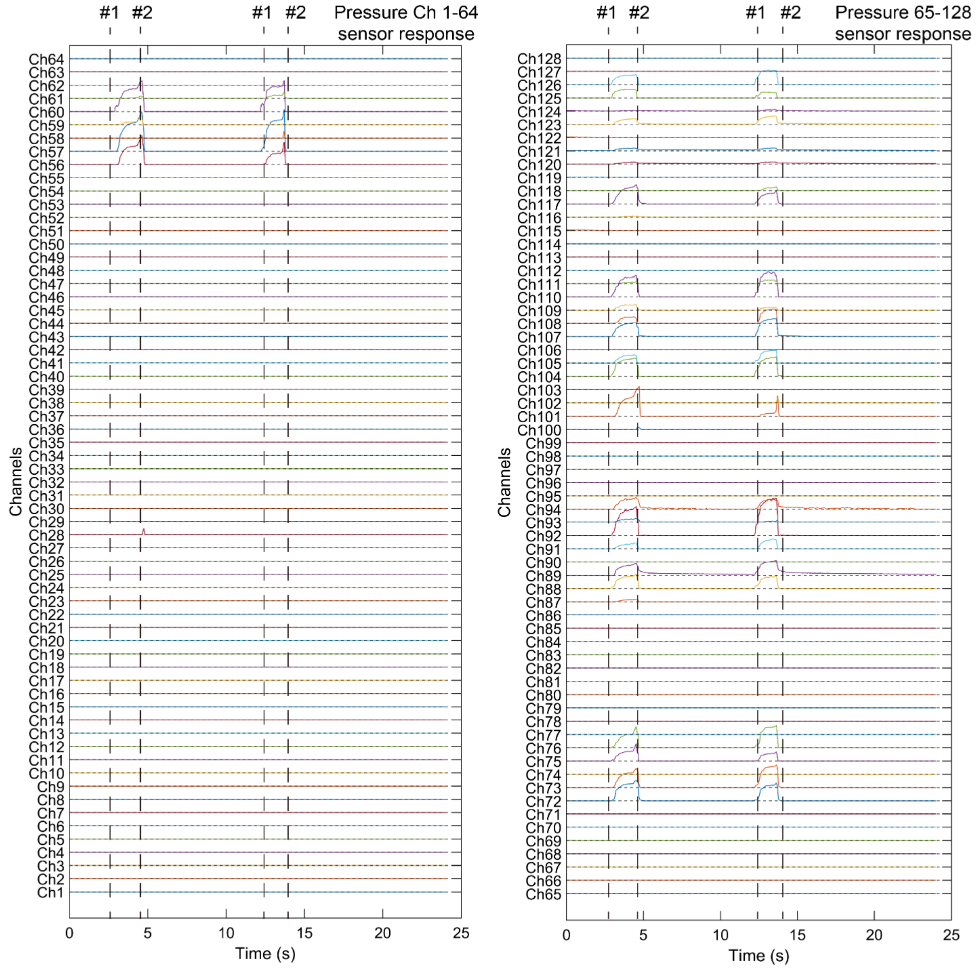

Supplementary Figure 21. Pressure time-series responses of channels 1-128 during partial-area hand slapping of the sensor array. #1 indicates the moment the hand makes contact, and #2 indicates the moment the hand is released.

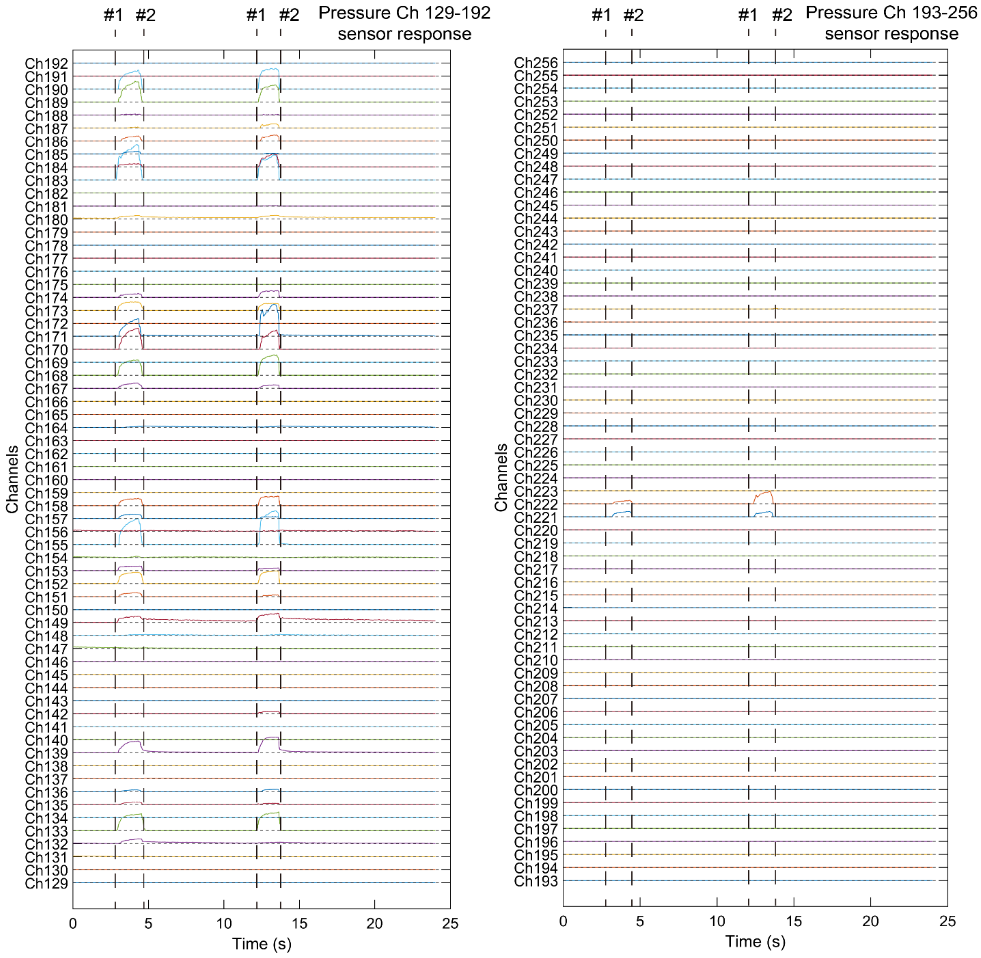

Supplementary Figure 22. Pressure time-series responses of channels 129-256 during partial-area hand slapping of the sensor array. #1 indicates the moment the hand makes contact, and #2 indicates the moment the hand is released.

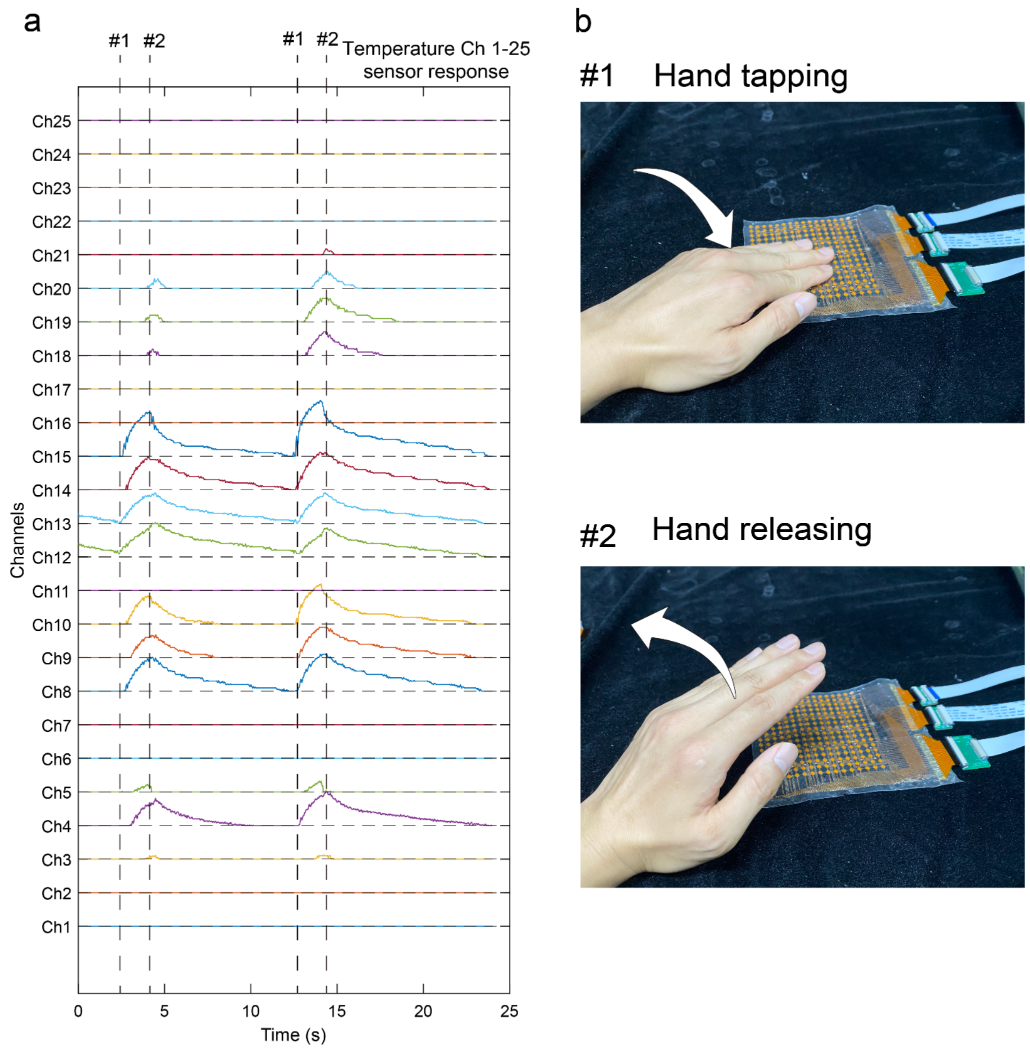

Supplementary Figure 23. Dynamic response recording of the sensor array. (a) Temperature time-series responses of channels 1-25 during partial-area hand slapping of the sensor array. **(b)** Description of the hand slapping sensor process. #1 indicates the moment the hand makes contact, and #2 indicates the moment the hand is released.

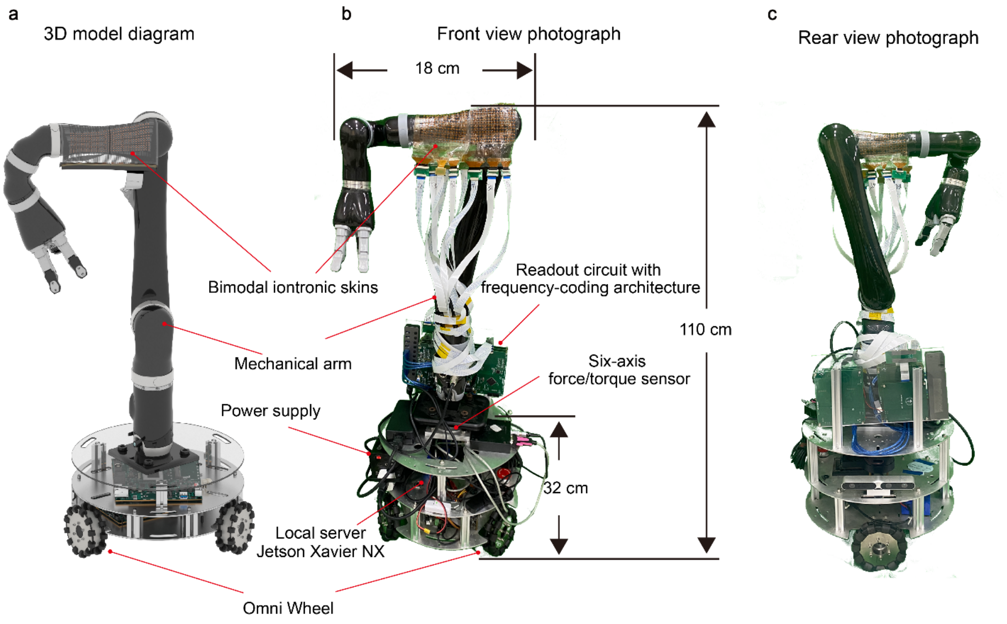

Supplementary Figure 24. Diagram of the collaborative robot's assembly components. (a) 3D model representation. Bimodal iontronic skins are integrated around the forearm section of the robotic arm to facilitate the user's support grip. The base layer comprises a power supply, readout circuit, six-axis force/torque sensor, and a local server. **(b)** Front-view photograph. **(c)** Rear-view photograph.

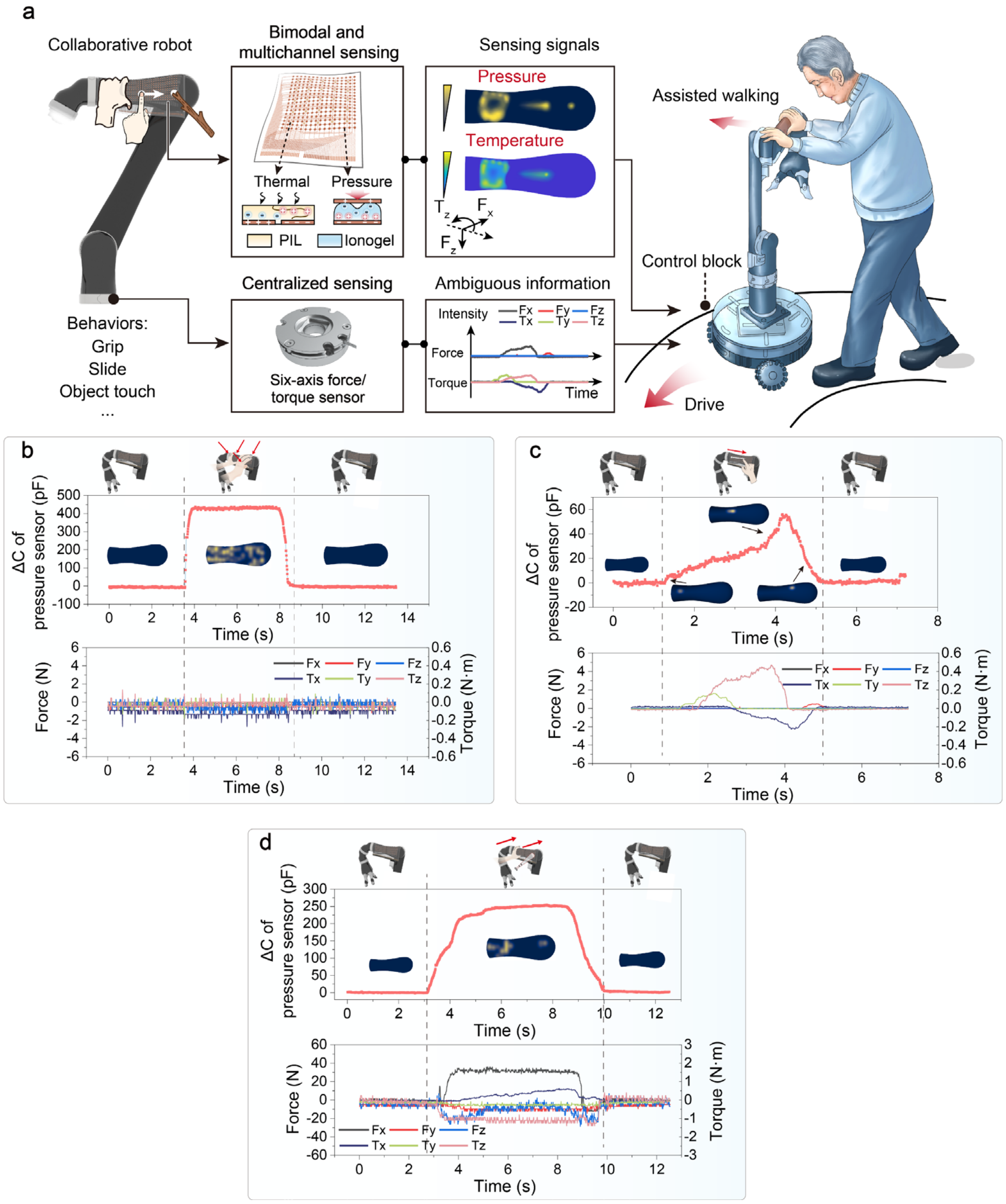

Supplementary Figure 25. Comparison of data responses between iontronic skins and end-effector (six-axis force/torque sensor) under different interaction motion modes. (a) Under gripping force, the spatial array points on the iontronic skins exhibit a significant response, whereas the six-axis force/torque sensors show minimal response due to force equilibrium. **(b)** During slight sliding touch, the iontronic skin array effectively records spatiotemporal signals, while the response from the six-axis force/torque sensors remains relatively weak. **(c)** Under different object and posture touches, the iontronic skin array captures distinct spatial features, whereas the six-axis force/torque sensors struggle to differentiate between various touch patterns.

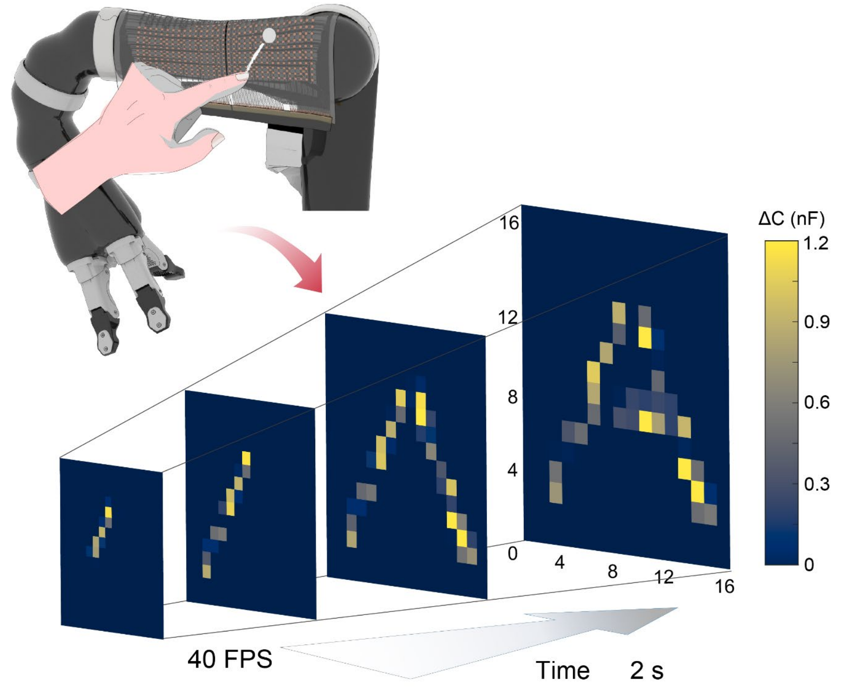

Supplementary Figure 26. Diagram of iontronic skins pressure recording for dynamic touch. It captures the spatiotemporal pressure variations during the dynamic writing of the letter 'A'.

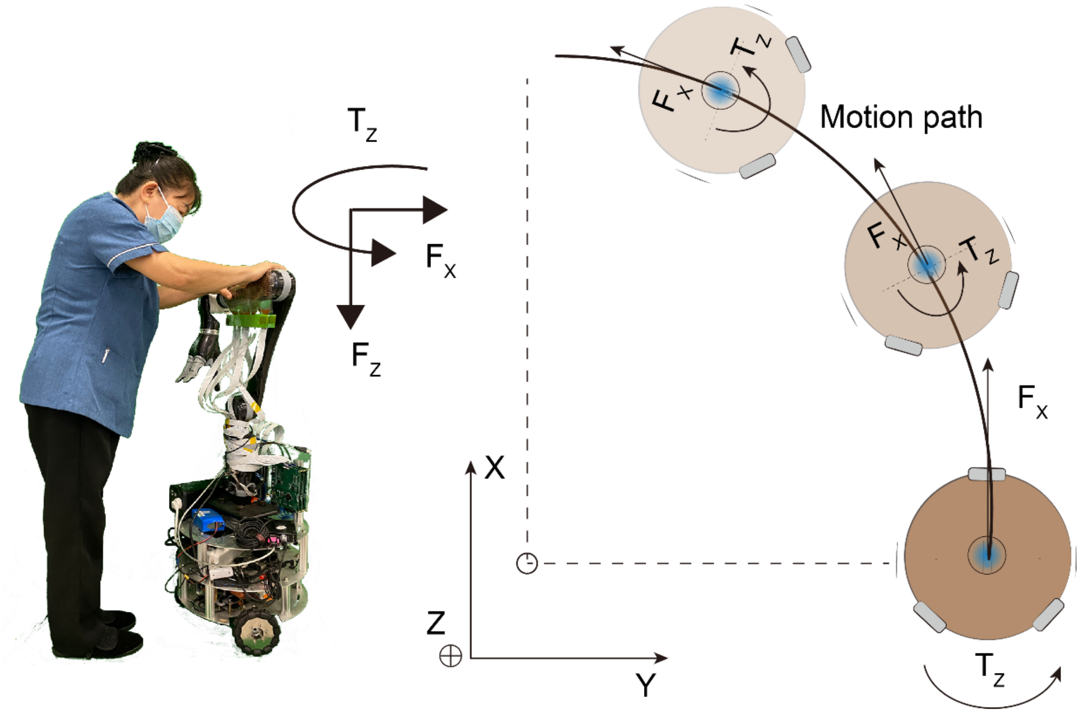

Supplementary Figure 27. Explanation of the apparent dynamics model. The apparent dynamics can be interpreted as the desired dynamics, wherein the robot's walking assistance behavior is guided by the user's intentions. The robotic system must dynamically adjust its linear and angular velocities in response to user-applied force/torque to achieve automatic gait synchronization, enabling continuous walking companionship.

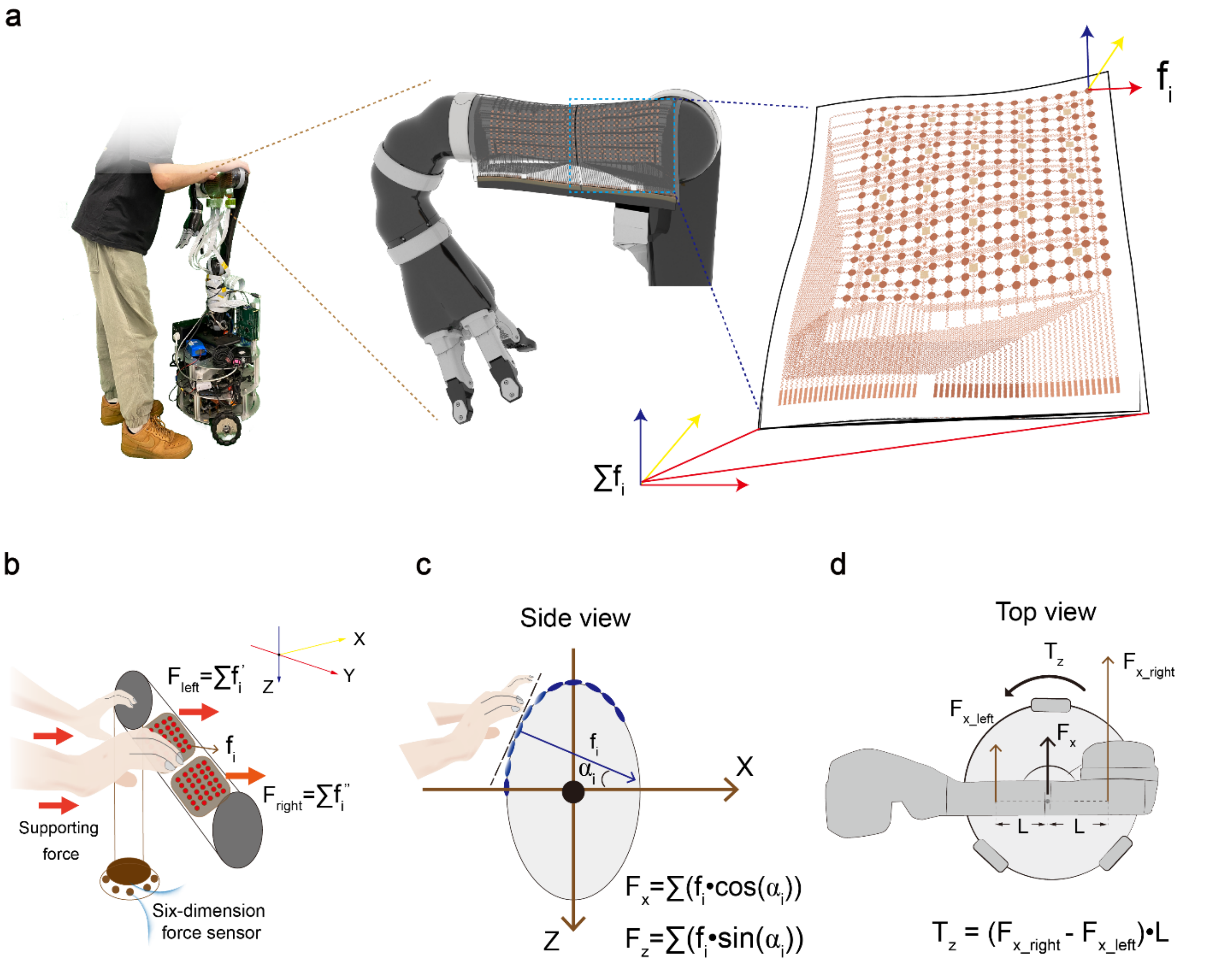

Supplementary Figure 28. Explanation of the vector mechanic model. (a) Schematic diagram of our iontronic skins framework. The resultant force on the surface of the flexible sensor is estimated as the approximate sum of the pressures measured by all the sensor units. **(b)** Approximate solution of the resultant force on the left and right handles. **(c)** Relying on the spatial positions of the sensor units within the iontronic skins wrapping model to map and decompose the interaction resultant force. **(d)** Torque estimation model in the collaborative robot.

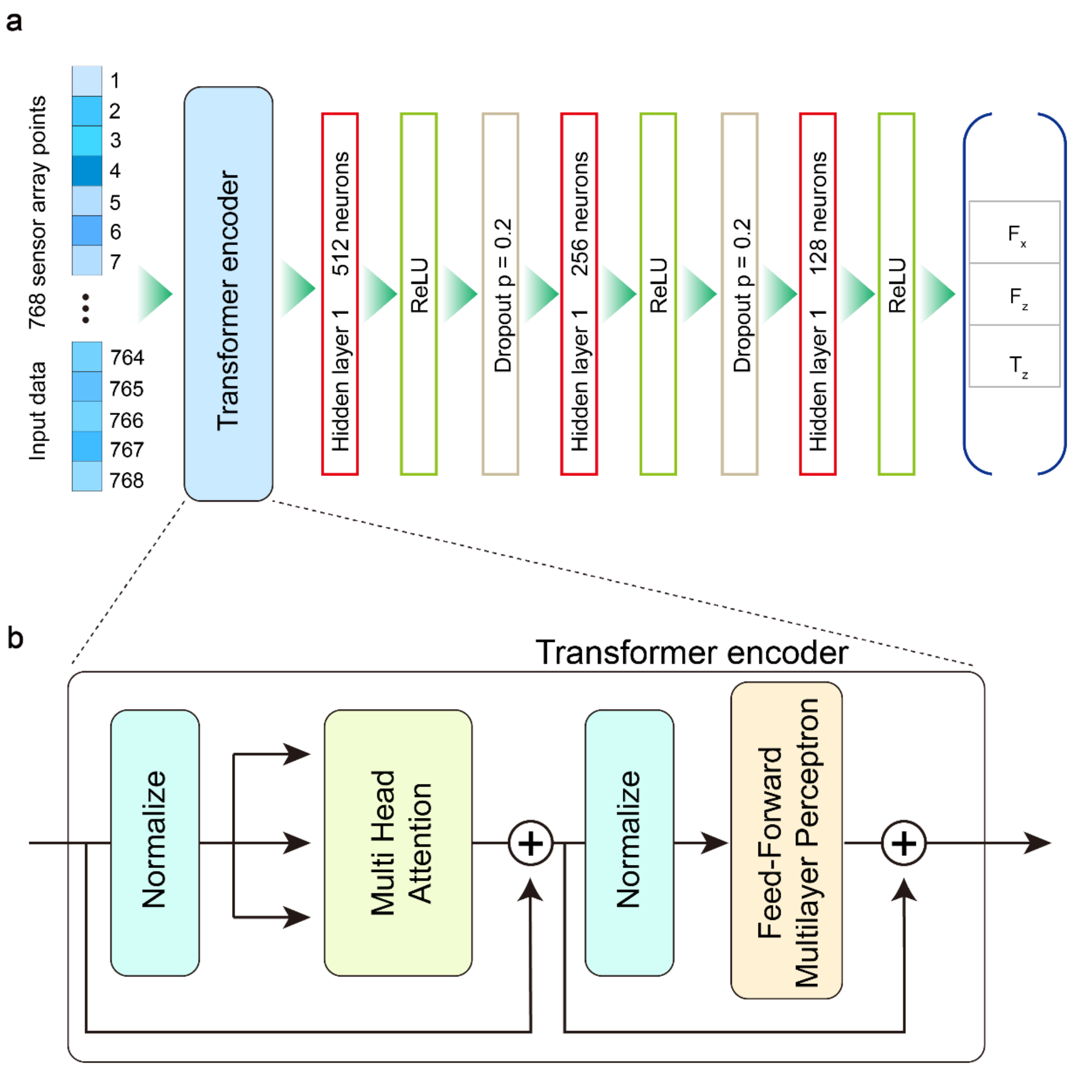

Supplementary Figure 29. Detailed description of the artificial neural network (ANN) architecture within the hybrid motion intention model (HMIM). (a) Schematic representation of the lightweight ANN structure, incorporating the MI-Transformer for learning features from both prior knowledge and real-time data. The MI-Transformer consists of a Transformer encoder and a multilayer perceptron, facilitating accurate inference and prediction of the assisted user's motion intention. This prediction is derived from the distributed somatosensory pressure data captured by the iontronic skins. **(b)** Detailed architecture of a Transformer encoder block. The input vectors first undergo a multi-head self-attention operation, followed by the first normalization layer, a feed-forward layer, and a second normalization layer. Skip connections, as indicated by the side arrows at the bottom, are incorporated to enhance gradient flow and model stability.

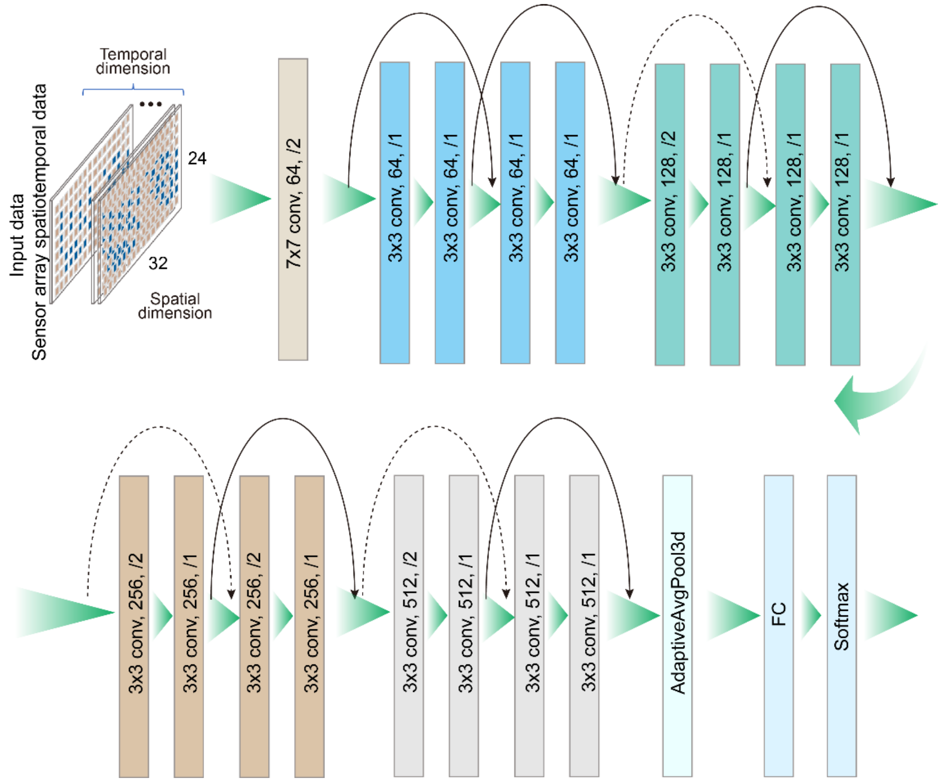

Supplementary Figure 30. ResNet-18 architecture. Downsampling strides are denoted as $w \times h$, where ($w \times h$) are spatial stride, respectively. Dotted lines are residual connections with downsampling.

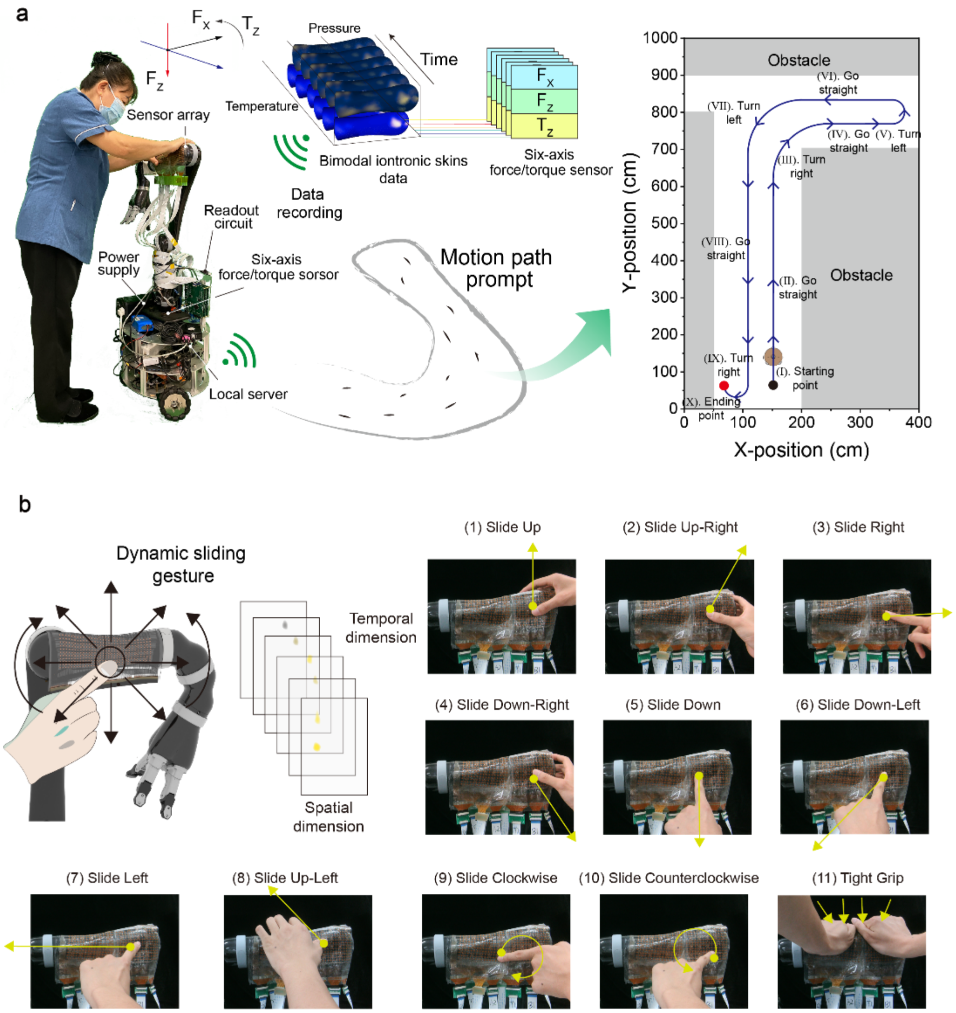

Supplementary Figure 31. Diagram illustrating the process of creating a dataset using bimodal iontronic skins. (a) The MI dataset includes bimodal iontronic skins and six-axis force/torque sensor data (recorded at 40 Hz over more than 200,000 frames) during simulations along various motion paths. The data was collected from 12 participants with different ages and movement habits (ages 20–54, 8 males, 4 females), with each participant completing four rounds of data collection while the robot remained stationary under high damping. In each round, participants simulate their control intentions by applying the expected control force to the robot based on the prompts of the motion path. The data from the iontronic skins is synchronized with the real-time data from the ground truth six-axis force/torque sensor. As shown in the figure on the right, the indicated path styles include different motion states, such as straight movement, large-radius left and right turns, small-radius left and right turns, and stationary states. This data is primarily used to correct the deviation between the iontronic skins data prediction and the six-axis force/torque sensor data. **(b)** The DG dataset comprises dynamic touch data recorded by iontronic skins from 12 participants. These participants performed 11 different dynamic touch gestures, including sliding motions in various directions and firm grips.

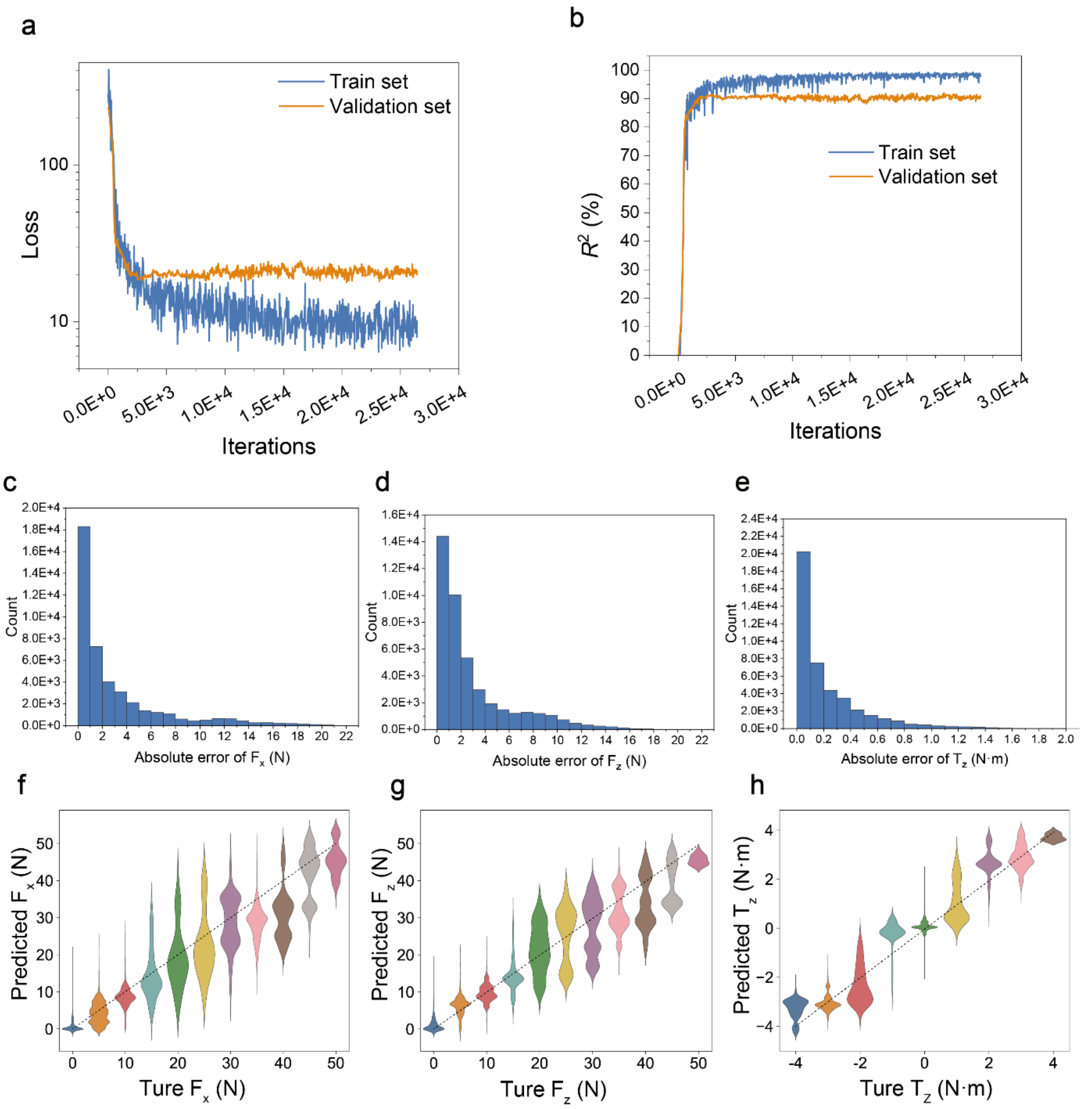

Supplementary Figure 32. Training of the HMIM for the MI prediction base on iontronic skins. (a) Loss convergence curve of the training. **(b)** R^2 convergence curve of the training. **(c)** Statistics of the absolute error of F_x. **(d)** Statistics of the absolute error of F_z. **(e)** Statistics of the absolute error of T_z. (**f** to **h**) MI inference test results based on the developed HMIM. The test dataset was constructed by experimentally testing samples under different motion intention paths from two subjects.

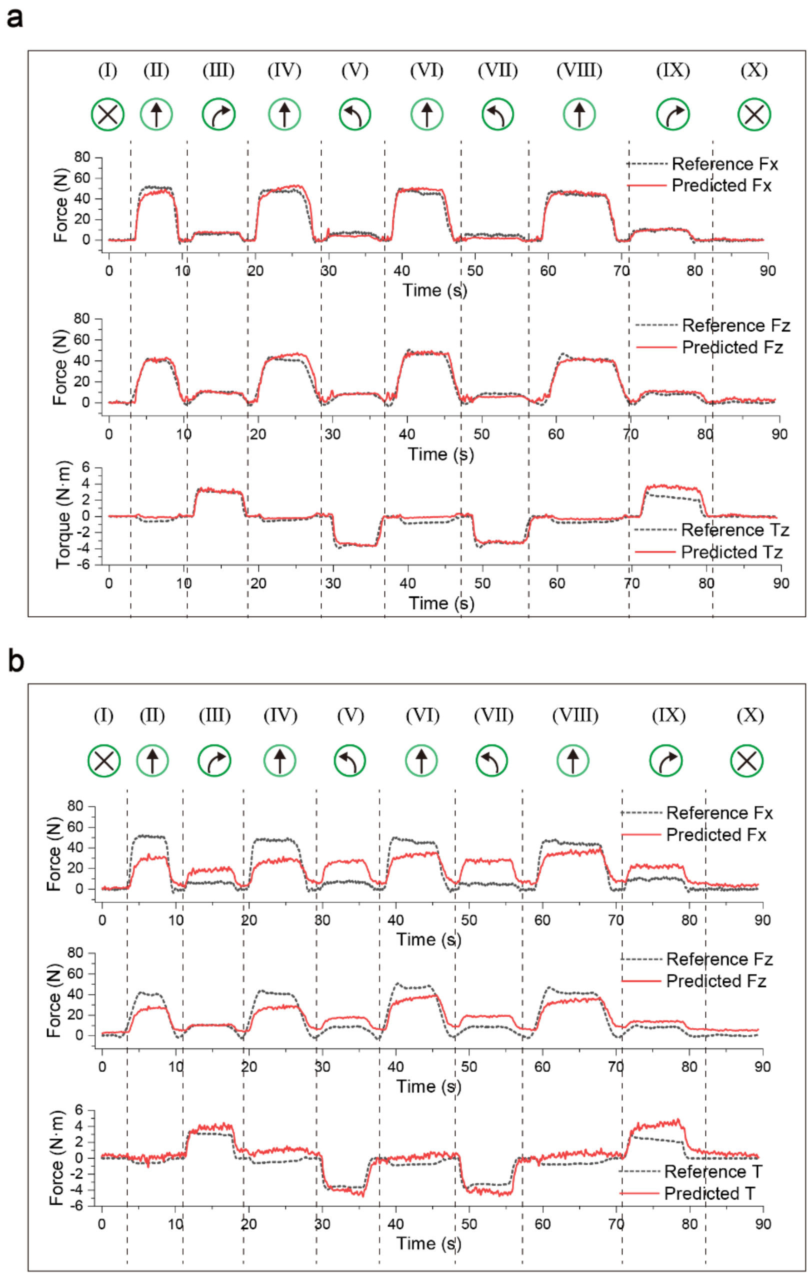

Supplementary Figure 33. (a) Comparison of MI inference results with the ground truth reference over a complete motion cycle during the HMIM inference testing. **(b)** Comparison of MI inference results with the ground truth reference over a complete motion cycle during the physical model VMM inference testing. The prediction of the physical model deviates from the actual ground truth due to sensor attachment errors and consistency errors in the sensing units.

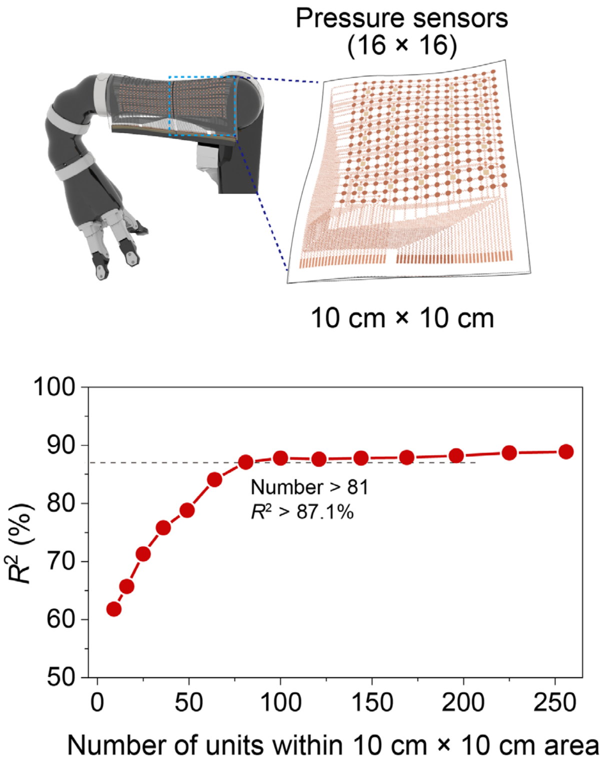

Supplementary Figure 34. The impact of the number of sensing units on inference accuracy. As the number of tactile perception units decreases, there is a significant drop in inference accuracy. When the number of units within a 10 cm × 10cm area exceeds 81, the inference accuracy starts gradually saturate.

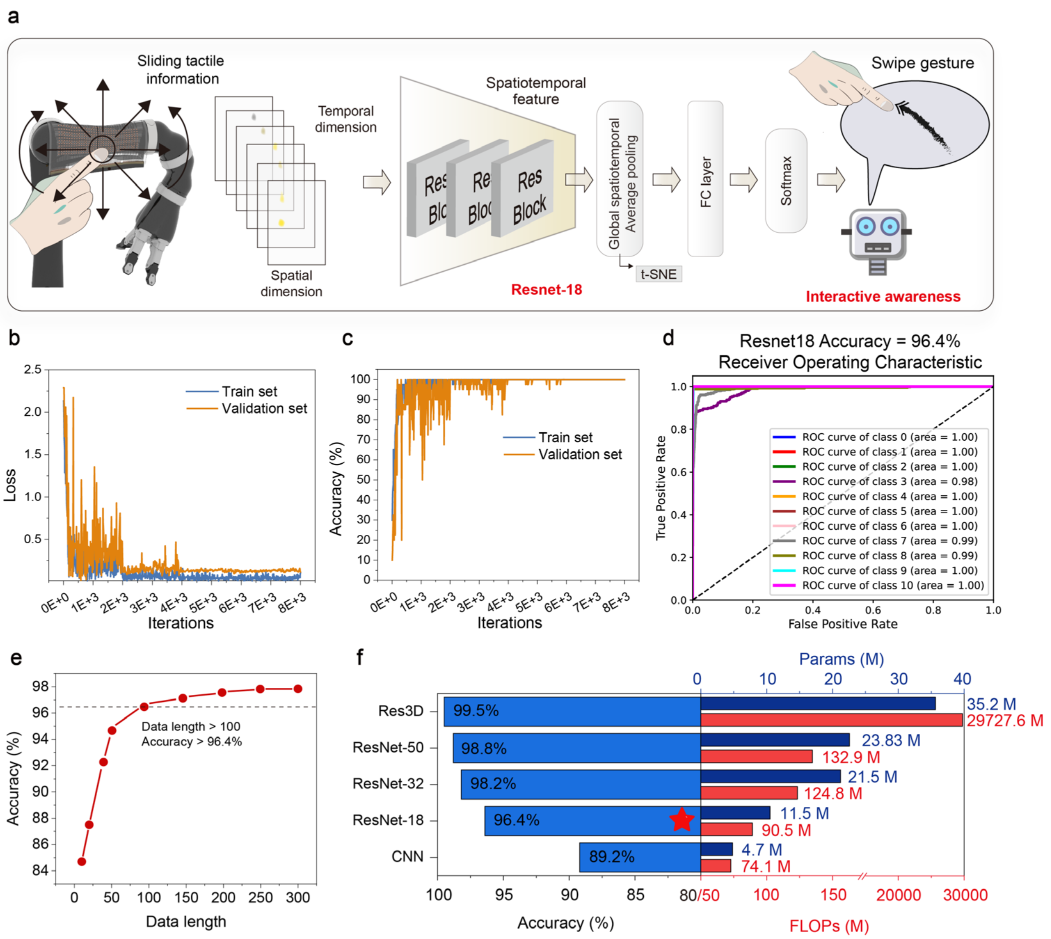

Supplementary Figure 35. Training of the DG recognition Resnet based on iontronic skins. **(a)** Resnet-18 network recognition process diagram (Time frame length=100). **(b)** Loss convergence curve of the training. **(c)** Accuracy convergence curve of the training. **(d)** The Receiver Operating Characteristic (ROC) curves of the Resnet-18 network for recognizing 11 types of dynamic touch gestures based on iontronic skins. **(e)** Recognition accuracy corresponding to different temporal frame lengths. **(f)** Comparison of mainstream spatiotemporal feature extraction networks. ResNet-18 achieves a balance between high recognition accuracy and computational efficiency.

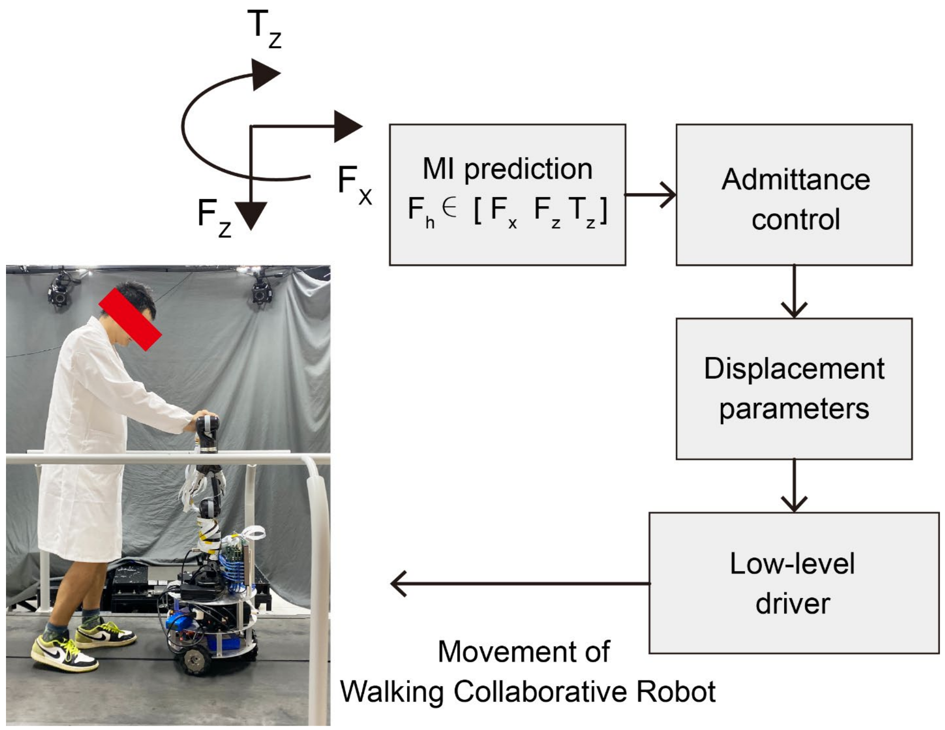

Supplementary Figure 36. A passive, intent-driven collaborative interaction paradigm. An admittance controller adjusts the linear and angular velocities of robot based on user-applied force/torque to achieve automatic gait synchronization, enabling continuous walking companionship.

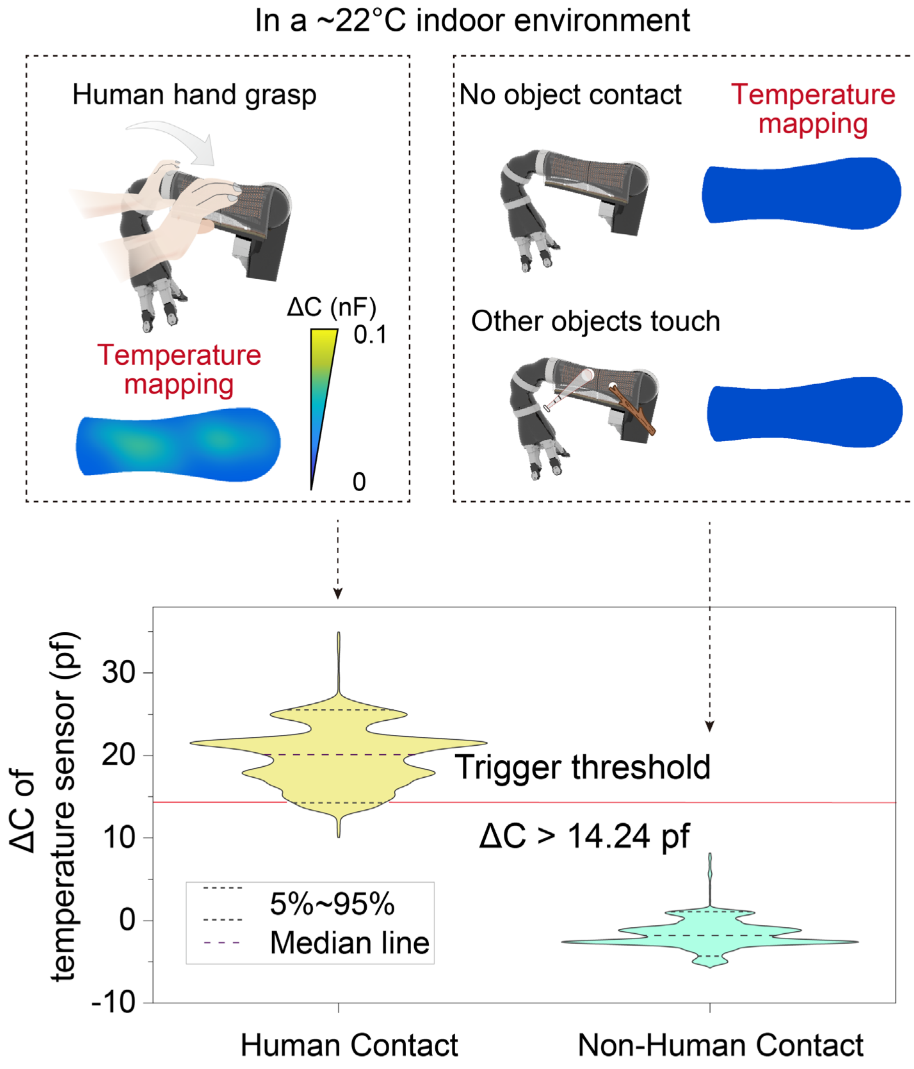

1

2 **Supplementary Figure 37. Temperature data distribution comparison between human hand**
3 **grasp and non-human touch conditions for 12 subjects (in a ~22°C indoor environment).** The
4 lowest 5th percentile of the human body temperature response is used as the threshold for
5 triggering motion control to ensure secure hand grasp collaborative interaction.

6

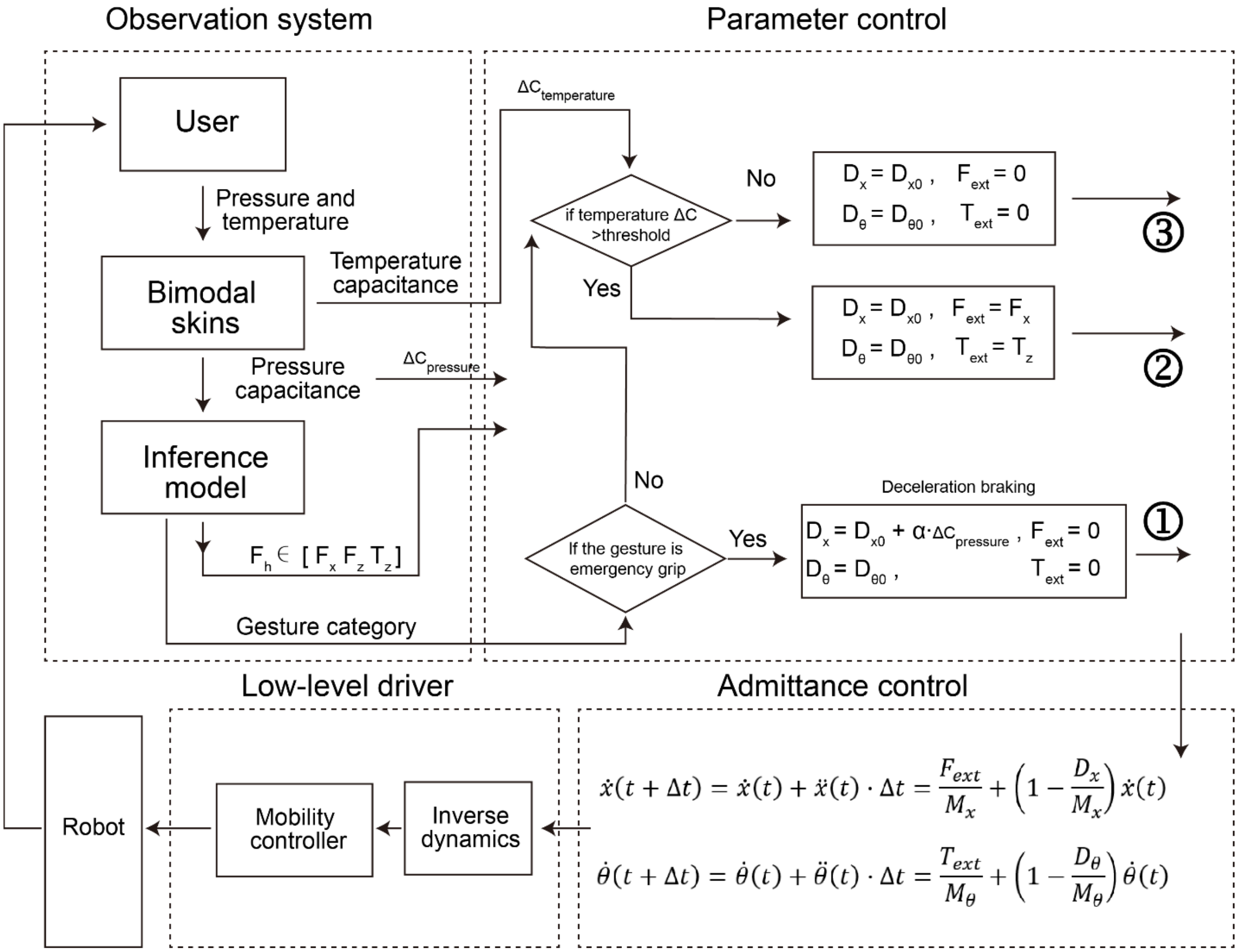

Supplementary Figure38. Flowchart of the robot motion control system. The system executes admittance control through edge-intelligent bimodal skin perception to ensure compliant walking collaboration and emergency braking detection.

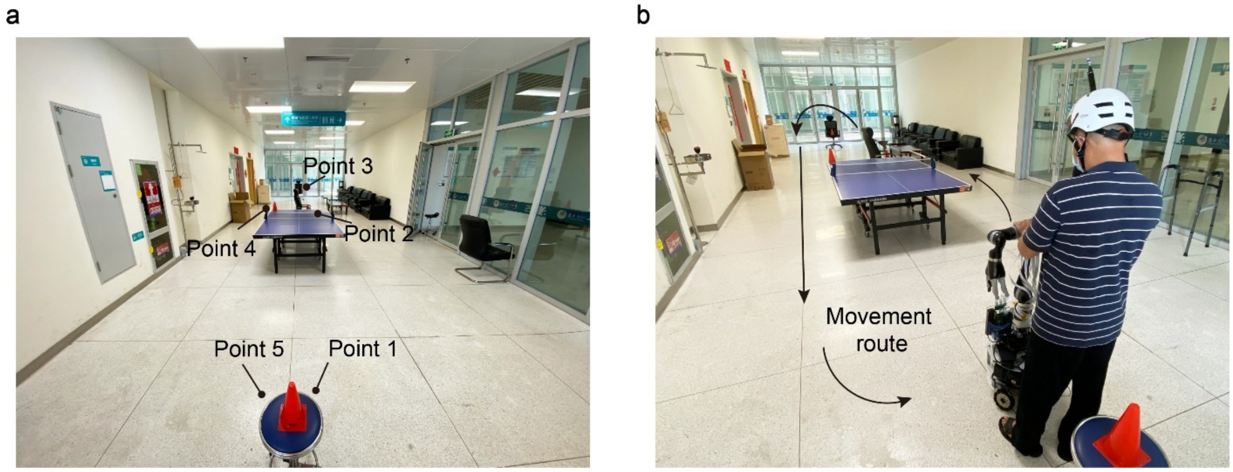

Supplementary Figure 39. The schematic diagram of the experimental site for assisted mobility of subject. (a) Position markers for the target points in the mobile experiment. **(b)** Photos of the actual movement path.

Supplementary Tables

Sensor type	Sensitivity	Pressure range	Strain insensitivity
Our Iontronic	11.5 kPa^{-1} (0-200 kPa) & 3.5 kPa^{-1} (200-520 kPa) & 1.1 kPa^{-1} (520-1000 kPa)	0-1 MPa	30%
Iontronic [13]	4.5 kPa^{-1} (0-1kPa) & 2.0 kPa^{-1} (1-10 kPa)	0-10 kPa	30%
Iontronic [14]	8.14kPa^{-1} (0-50 kPa) & 1.23 kPa^{-1} (50-250 kPa)	0-250 kPa	15%
Iontronic [15]	1.92 kPa^{-1} (0-2 kPa) & 0.74 kPa^{-1} (2-10 kPa)	0-10 kPa	50%
Iontronic [12]	810kPa^{-1} (0-50 kPa) & 364 kPa^{-1} (50-200 kPa) & 174 kPa^{-1} (200-400 kPa)	400 kPa	30% (sensitive to strain)
Iontronic [16]	2.47 kPa^{-1}	0-2 MPa	20%
Pizoresistive [17]	31.6 kPa^{-1}(0-0.25 kPa) & 10.61 kPa^{-1} (1-50 kPa)	0-50 kPa	20%
Pizoresistive [18]	0.01 kPa^{-1}	0-250 kPa	30% (sensitive to strain)
Pizoresistive [19]	888.79 kPa^{-1} (0-20 kPa) & 355.56 kPa^{-1} (20-100 kPa)	0-100 kPa	30%
Pizoresistive [20]	6 N^{-1} (0-0.1 N) & 0.15 N^{-1} (0.1-2 N)	0-2 N	100% (sensitive to strain)
Pizoresistive [21]	0.17 kPa^{-1}	0-40 kPa	30%
Pizoresistive [22]	4.88 kpa^{-1}	0-8 kPa	30% (sensitive to strain)
Pizoresistive [23]	10^6 kPa^{-1}	0-1 kpa	7% bending strain
Triboelectric [24]	4.81 kPa^{-1} (0-2 kPa) & 1.96 kPa^{-1} (2-10 kPa)	0-10 kPa	50%
Triboelectric [25]	118.18 $mV \cdot kPa^{-1}$ (0-10 kPa) & 28.31 $mV \cdot kPa^{-1}$ (10-150 kPa)	0-150 kPa	70%
Capacitive [26]	0.277 kPa^{-1} (0-1kPa) & 0.064 kPa^{-1} (1-8 kPa)	0-8 kPa	70%
Capacitive [27]	0.23 kPa^{-1}	0-1 MPa	50% (sensitive to strain)
Capacitive [28]	0.7 kPa^{-1} (0-5 kPa) & 0.13 kPa^{-1} (5-10 kPa)	0-10 kPa	15% (sensitive to strain)
Magnetic [29]	0.2 $\mu A \cdot kPa^{-1}$	0-120 kPa	530%

Supplementary Table 1. The sensing performances of stretchable pressure senor comparison.

Method	Intrinsic force sensing	Pressure resolution	Readout rate	Sensing channels	Pressure range
Six-axis force/torque (SARA)[30,31]	Relies on complex deep learning models to infer intrinsic force information.	~0.1 N	8K FPS	6	~10 kPa
Six-axis force/torque (UR10e)[32]	No	5 N	~500 FPS	6	~ 10 kPa
Six-axis force/torque (RFT64-6A01)[33]	Intrinsic force sensing modeling is required.	0.08 N	1K FPS	6	~ 10 kPa
Six-axis force/torque[3]	No	--	--	6	--
Our iontronic skins	Yes	0.01 N	93 FPS	768	1000 kPa

Supplementary Table 2. A comparative analysis of the application performance of our flexible iontronic skins and rigid end-effector (six-axis force/torque sensors) for robotic tactile perception. Compared to the end-effector, our skin offers distinct advantages, including intrinsic force sensing, high-pressure resolution, multidimensional feature detection, and a wide pressure sensing range.

**Lightweight neural network**	R^2 (F_x)	R^2 (F_z)	R^2 (T_z)	R^2 **(Average)**	**Params**	**FLOPs**	**Jetson Xavier NX-16GB**
CNN	83.2%	84.3%	81.2%	82.9%	6.37M	216.43M	39.3 ms
ResNet-18 [34]	86.7%	86.4%	85.1%	86.1%	11.17M	87.36M	16.2 ms
MobileNetV2 [35]	73.1%	72.1%	79.4%	74.9%	2.23M	5.62M	6.7 ms
ShuffleNet [36]	83.1%	83.2%	52.8%	73.0%	1.26M	2.69M	2.6 ms
MobileViT [37]	89.4%	86.2%	84.5%	86.7%	3.37M	3.37M	3.6 ms
MI-Transformer	88.9%	88.8%	88.3%	88.7%	3.32M	3.31M	3.2 ms

Supplementary Table 3. Comparison of performance results for lightweight intent prediction neural networks. In comparison, our MI-Transformer achieves higher prediction accuracy with lightweight computational requirements.

**Sensor type**	**Sensing task**	**Model architecture**	**FLOPs**	**Edge device**	**Reference**
IMU, EMG	Gesture prediction	CNN	~ 100M	×	*Nature Sensors* (2025): 1-13. [38]
Iontronic array	Object recognition	1D-CNN	~ 60M	×	*Science Advances*, 2023, 9(9): eadf8831. [12]
Iontronic array	Touch recognition	Res3D	> 1000M	×	*NPJ Flexible Electronics* 8.1 (2024): 9. [1]
Hydrogel-based sensor array	Posture recognition	ResNet-18/Random Forest	>90M	×	*Adv. Funct. Mater.*, e19562 (2025). [39]
Piezoresistive array	Posture recognition	CompHVE	--	✓	*Research 7, 0497 (2024).* [40]
Multimodal sensors	Robotic decision-making and obstacle avoidance	Hyperdimensional computing	--	✓	*Nat Commun* **16**, 8818 (2025). [41]
Our bimodal iontronic skins	Motion intent prediction and robotic decision-making	MI-Transformer & ResNet-18	93.6M	✓	Our work

Supplementary Table 4. AI-enhanced sensing systems exhibit fundamentally different task distributions when deployed on edge devices versus cloud/PC platforms. Cloud- or PC-based implementations are mainly responsible for computationally intensive model training, large-scale data aggregation, and offline analysis, which can utilize rich centralized resources but increase latency, bandwidth consumption, and potential privacy exposure [42]. In contrast, edge-based implementations emphasize lightweight system design and on-device inference, enabling real-time intelligence and seamless interaction between users and their environments [43]. By pushing deep learning inference closer to the data source, edge intelligence substantially reduces end-to-end latency and communication overhead while enhancing privacy preservation and system robustness. Consequently, for AI-enhanced sensing and control scenarios that are highly sensitive to timeliness, reliability, and security, edge intelligence is not merely advantageous but essential.

Supplementary Movie 1.

Real-time response demonstration of bimodal iontronic skins

Supplementary Movie 2.

Real-time multisensory response demonstration of our robot.

Supplementary Movie 3.

Pressure response of the robot under different touch actions.

Supplementary Movie 4.

Real-time motion intention prediction using the hybrid motion intention model (HMIM) and the vector mechanics model (VMM).

Supplementary Movie 5.

Demonstration of a motion control game based on iontronic skins.

Supplementary Movie 6.

Demonstration of providing mobility assistance to individuals with movement impairments.

References

1. Li, Z., Yang, J., Zhang, Y. et al. Ultrafast readout, crosstalk suppression iontronic array enabled by frequency-coding architecture. *npj Flex. Electron.* **8**, 9 (2024).

2. Kalinowska, A., Pilarski, P.M. &Murphey, T.D. Embodied communication: How robots and people communicate through physical interaction. *Annu. Rev. Control Rob. Auton. Syst.* **6**, 205-232 (2023).

3. Chuy, O.Y., Hirata, Y., Wang, Z. et al. A control approach based on passive behavior to enhance user interaction. *IEEE Trans. Rob.* **23**, 899-908 (2007).

4. Andreetto, M., Divan, S., Ferrari, F. et al. Simulating passivity for robotic walkers via authority-sharing. *IEEE Rob. Autom. Lett.* **3**, 1306-1313 (2018).

5. Chuy, O., Hirata, Y. &Kosuge, K. in 2004 IEEE/RSJ International Conference on Intelligent Robots and Systems (IROS)(IEEE Cat. No. 04CH37566), Vol. 3 2289-2294 (IEEE, 2004).

6. Cirillo, A., Ficuciello, F., Natale, C. et al. A conformable force/tactile skin for physical human–robot interaction. *IEEE Rob. Autom. Lett.* **1**, 41-48 (2015).

7. Fairbanks, B.D., Love, D.M. &Bowman, C.N. Efficient Polymer‐Polymer Conjugation via Thiol‐ene Click Reaction. *Macromol. Chem. Phys.* **218**, 1700073 (2017).

8. Decker, C. &Decker, D. Photoinitiated radical polymerization of vinyl ether-maleate systems. *Polymer* **38**, 2229-2237 (1997).

9. McHugh, P.J., Das, A.K., Wallace, A.G. et al. An Investigation of a (vinylbenzyl) trimethylammonium and N-vinylimidazole-substituted poly (vinylidene fluoride-co-hexafluoropropylene) copolymer as an anion-exchange membrane in a lignin-oxidising electrolyser. *Membranes* **11**, 425 (2021).

10. Mukhopadhyay, S., Sahu, P., Bhajiwala, H. et al. Synthesis, characterization and properties of self-healable ionomeric carboxylated styrene–butadiene polymer. *J. Mater. Sci.* **54**, 14986-14999 (2019).

11. Wong, D.H., Thelen, J.L., Fu, Y. et al. Nonflammable perfluoropolyether-based electrolytes for lithium batteries. *Proceedings of the National Academy of Sciences* **111**, 3327-3331 (2014).

12. Shi, J., Dai, Y., Cheng, Y. et al. Embedment of sensing elements for robust, highly sensitive, and cross-talk–free iontronic skins for robotics applications. *Sci. Adv.* **9**, eadf8831 (2023).

13. Su, Q., Zou, Q., Li, Y. et al. A stretchable and strain-unperturbed pressure sensor for motion interference–free tactile monitoring on skins. *Sci. Adv.* **7**, eabi4563 (2021).

14. Wang, P., Liu, J., Yu, W. et al. Flexible, stretchable, breathable and sweatproof all-nanofiber iontronic tactile sensor for continuous and comfortable knee joint motion monitoring. *Nano Energy* **103**, 107768 (2022).

15. Liu, X., Ji, X., Zhu, R. et al. A microphase‐separated design toward an all‐round ionic hydrogel with discriminable and anti‐disturbance multisensory functions. *Adv. Mater.* **36**, 2309508 (2024).

16. Shi, J., Xie, S., Liu, Z. et al. Non-hygroscopic ionogel-based humidity-insensitive iontronic sensor arrays for intra-articular pressure sensing. *Natl. Sci. Rev.* **11**, nwae351 (2024).

17. Lou, Z., Chen, S., Wang, L. et al. Ultrasensitive and ultraflexible e-skins with dual functionalities for wearable electronics. *Nano Energy* **38**, 28-35 (2017).

18. Gerratt, A.P., Michaud, H.O. &Lacour, S.P. Elastomeric electronic skin for prosthetic tactile sensation. *Adv. Funct. Mater.* **25**, 2287-2295 (2015).

19. Choi, S.B., Noh, T., Jung, S.B. et al. Stretchable Piezoresistive Pressure Sensor Array with Sophisticated Sensitivity, Strain‐Insensitivity, and Reproducibility. *Adv. Sci.* **11**, 2405374 (2024).

20. Ge, J., Sun, L., Zhang, F.R. et al. A stretchable electronic fabric artificial skin with pressure‐, lateral strain‐, and flexion‐sensitive properties. *Adv. Mater.* **28**, 722-728 (2016).

21. Roh, E., Lee, H.B., Kim, D.I. et al. A solution‐processable, omnidirectionally stretchable, and high‐pressure‐sensitive piezoresistive device. *Adv. Mater.* **29**, 1703004 (2017).

22. Choong, C.L., Shim, M.B., Lee, B.S. et al. Highly stretchable resistive pressure sensors using a conductive elastomeric composite on a micropyramid array. *Adv. Mater.* **26**, 3451-3458 (2014).

23. Lee, S., Reuveny, A., Reeder, J. et al. A transparent bending-insensitive pressure sensor. *Nat. Nanotechnol.* **11**, 472-478 (2016).

24. He, J., Zhou, R., Zhang, Y. et al. Strain‐insensitive self‐powered tactile sensor arrays based on intrinsically stretchable and patternable ultrathin conformal wrinkled graphene‐elastomer composite. *Adv. Funct. Mater.* **32**, 2107281 (2022).

25. Zhang, L., Gao, Z., Lei, H. et al. Strain-insensitive stretchable triboelectric tactile sensors via interfacial stress dispersion. *Nano Energy* **133**, 110482 (2025).

26. Ma, C., Wang, M., Wang, K. et al. Ultrasensitive, highly selective, integrated multidimensional sensor based on a rigid-flexible synergistic stretchable substrate. *Adv. Fiber Mater.* **5**, 1392-1403 (2023).

27. Lipomi, D.J., Vosgueritchian, M., Tee, B.C. et al. Skin-like pressure and strain sensors based on transparent elastic films of carbon nanotubes. *Nat. Nanotechnol.* **6**, 788-792 (2011).

28. Boutry, C.M., Kaizawa, Y., Schroeder, B.C. et al. A stretchable and biodegradable strain and pressure sensor for orthopaedic application. *Nat. Electron.* **1**, 314-321 (2018).

29. Xu, J., Duan, C., Wan, X. et al. A soft magnetoelastic sensor to decode levels of fatigue. *Nat. Electron.* **8**, 709-720 (2025).

30. Iskandar, M., Albu-Schäffer, A. &Dietrich, A. Intrinsic sense of touch for intuitive physical human-robot interaction. *Sci. Rob.* **9**, eadn4008 (2024).

31. Iskandar, M., Ott, C., Eiberger, O. et al. in 2020 IEEE/RSJ International Conference on Intelligent Robots and Systems (IROS) 8903-8910 (IEEE, 2020).

32. Bolli, R., Bonato, P. &Asada, H.H. in 2023 IEEE/RSJ International Conference on Intelligent Robots and Systems (IROS) 122-129 (IEEE, 2023).

33. Kim, U., Jo, G., Jeong, H. et al. A novel intrinsic force sensing method for robot manipulators during human–robot interaction. *IEEE Trans. Rob.* **37**, 2218-2225 (2021).

34. He, K., Zhang, X., Ren, S. et al. in Proceedings of the IEEE conference on computer vision and pattern recognition 770-778 (2016).

35. Sandler, M., Howard, A., Zhu, M. et al. in Proceedings of the IEEE conference on computer vision and pattern recognition 4510-4520 (2018).

36. Zhang, X., Zhou, X., Lin, M. et al. in Proceedings of the IEEE conference on computer vision and pattern recognition 6848-6856 (2018).

37. Mehta, S. &Rastegari, M. Mobilevit: light-weight, general-purpose, and mobile-friendly vision transformer. *arXiv preprint arXiv:2110.02178* (2021).

38. Chen, X., Lou, Z., Gao, X. et al. A noise-tolerant human–machine interface based on deep learning-enhanced wearable sensors. *Nature Sensors*, 1-13 (2025).

39. Wang, L., Guo, X., Zhang, Z. et al. Metaverse‐Enabled Yoga Coach Avatar Using AI‐Enhanced Multimodal Insole Sensing System. *Adv. Funct. Mater.*, e19562 (2025).

40. Xu, Z., Zhang, F., Xie, E. et al. A flexible, large-scale sensing array with low-power in-sensor intelligence. *Research* **7**, 0497 (2024).

41. Li, J., Xu, Z., Li, N. et al. AI-embodied multi-modal flexible electronic robots with programmable sensing, actuating and self-learning. *Nat. Commun.* **16**, 8818 (2025).

42. Ficili, I., Giacobbe, M., Tricomi, G. et al. From sensors to data intelligence: Leveraging IoT, cloud, and edge computing with AI. *Sensors* **25**, 1763 (2025).

43. Guo, X., Zhang, Z., Ren, Z. et al. Advances in Intelligent Nano‐Micro‐Scale Sensors and Actuators: Moving toward Self‐Sustained Edge AI Microsystems. *Adv. Mater.* **37**, e10417 (2025).
